# When Can Orthodontic Over-Treatment Be Measured? A Pre-Registered Identification Assessment, a Falsified Claim, and a Cross-Sectional Analysis of Untreated Occlusal Need in NHANES III

**DOI:** 10.3390/healthcare14203395

**Published:** 2026-10-12

**Authors:** Ahmed Mohammed Alassiry

**Affiliations:** Department of Preventive Dental Sciences, Faculty of Dentistry, Najran University, P.O. Box 1988, Najran 55461, Saudi Arabia; amyahia@nu.edu.sa

**Keywords:** malocclusion, orthodontics, health services needs and demand, healthcare disparities, health policy, Bayesian analysis, medical overuse

## Abstract

**Highlights:**

**What are the main findings?**

- A pre-registered falsification test on four identification conditions fired: US National Health Examination Survey Cycles II and III (1963–70), two individually linked cycles in the open public domain, satisfy all four. The claim that no currently available open-data source identifies orthodontic over-treatment is withdrawn. Of 26 candidate sources assessed, 25 fail, and they fail in the two families the framework distinguishes—population surveys on I1, administrative sources on I3.
- Among US children aged 8–17, the weighted prevalence of occlusal traits at or above the specified severity thresholds ranged from 23.4% to 44.5% across four composites, and 87.8% of those with need had never been treated, with untreated occlusal need falling 15.9 pp from the most to the least disadvantaged income rank.

**What are the implications of the main findings?**

- Below-threshold shares taken from need-gated administrative records measure enforcement of the eligibility rule rather than clinical judgement, and carry no information about where the threshold should sit—but they are not the only possible evidence, and a universal negative over all sources does not follow.
- Closing the measurement gap does not require new data infrastructure: it requires repeat examination of the same children before and after the treatment age, on a population frame, with an instrument carrying no entitlement consequence. That design existed in the 1960s and has not been repeated.

**Abstract:**

**Background/Objectives**: Orthodontic care is widely described as simultaneously over-supplied to those with little clinical need and under-supplied to those with much. Testing the first half of that claim requires a data source in which need is recorded independently of entitlement. We asked whether any such source exists, and, where need can be measured independently, how much of it remains untreated and how that residual is distributed socially. **Methods**: We pre-registered four conditions under which population-level over-treatment is identified from a data source: need measured before treatment on the same individuals (I1); a sampling frame containing treated and untreated individuals (I2); need measurement not conditioned on treatment eligibility or reimbursement (I3); and treatment receipt observed irrespective of payer (I4). The pre-registered falsification rule was that if any source satisfied all four, the central claim would be reported as falsified. At reviewers’ request, we replaced the original four-source convenience assessment with a documented search: 112 requests logged across eight frames, 15,341 records identified, and 14,139 records screened. Residual untreated occlusal need and its social gradient were estimated in a cross-sectional analysis of the Third National Health and Nutrition Examination Survey (NHANES III, 1988–1994) using a design-informed Bayesian hierarchical logistic model, cross-checked against Taylor-linearised design-based estimation. **Results**: The falsification rule fired. Of 26 sources assessed against the four conditions, 25 fail—and their failures fall into the two families the framework distinguishes: administrative sources on I3 and population surveys on I1—but one satisfies all four. The US National Health Examination Survey Cycles II (1963–65) and III (1966–70) do so because the two cycles are individually linked, giving a pre-adolescent occlusal record on 2177 individuals in the adolescent sample; excluding those already in appliance therapy at Cycle II leaves 2075 individuals. The source is open. Estimand E2 is therefore falsified, and the universal claim is withdrawn; the mechanism it describes is not. In NHANES III, weighted need prevalence among 4593 children aged 8–17 ranged from 23.4% to 44.5% across four composites—the range is the finding, and the point estimate is secondary to it—with 33.3% (31.4% to 35.2%) under the registered composite. Of children with need present, 87.8% had never been treated. Standardised risk of untreated need—a population-standardised posterior quantity, not an observed proportion—fell from 37.6% at the most disadvantaged income rank to 21.7% at the most advantaged: a risk difference of 15.9 pp (11.1 pp to 20.6 pp). The gradient survives a differential report-error correction up to 2.0× and agrees with design-based estimation to within 0.046 pp. **Conclusions**: Over-treatment is not identified by the need-gated administrative records from which it is usually estimated; the reason is structural: eligibility rules that respond to perceived over-treatment destroy the evidence of what they detect. It is identified by a linked open survey fielded sixty years ago, which is why the stronger claim that no such source exists does not survive. Untreated occlusal need, by contrast, is measurable, substantial, and socially graded.

## 1. Introduction

Two claims about orthodontic care are made so often that they are rarely examined together [1]. The first is that a portion of delivered treatment is clinically unnecessary [2,3,4]. The second is that people with the greatest clinical need are the least likely to be treated [5]. Both are plausible, both have empirical support, and they are usually presented as two faces of a single misallocation [1].

The first claim has a long evidential history. Turbill and colleagues scored a random sample of completed orthodontic cases from a national payment board and reported rates of treatment that did not meet the funder’s own criteria [2,3]. A federal audit of a state Medicaid programme found that a majority of a stratified random sample of approved prior authorisations did not qualify under the programme’s own rules [4]. The framing of two-sided misallocation—underuse among deprived groups alongside unnecessary care among affluent ones—is stated explicitly in the field’s flagship series [1].

What is being measured: We use over-treatment throughout in a specific, population-level sense: treatment delivered to individuals in whom, before treatment, normative clinical need was absent or below the severity at which the treatment is indicated. Its estimand is a prevalence within the treated population in the numerator and a population frame in the denominator. That is a different quantity from inappropriate treatment among recipients—the share of delivered cases that fail a funder’s criteria—which is what the audit literature above measures and which a treated-only record can support. The two are often reported interchangeably. They are not interchangeable, and the distinction is the whole subject of this paper.

What has received less attention is the measurement position from which the population-level claim can be made at all. Each of the studies above draws its evidence from a record generated by a payment system, and in every such system a need score exists because it determines entitlement. That is not a defect of any particular dataset. It is the purpose of the score. The consequence is that the distribution of need visible in the record is the distribution of need among those the rule admitted, which is not the distribution of need in the population, and is not the distribution that a population-level claim about over-treatment refers to.

This paper takes that observation seriously enough to formalise it. We state the conditions under which population-level over-treatment is identified from a data source, and test whether any openly available source satisfies them. We treat the result as falsifiable, and we report it as having been falsified: one source does satisfy all four, and the claim that none does is withdrawn.

The four conditions are this paper’s proposal, not an established framework, and they were not fixed before the study’s first sources were inspected. Section 2.1 and Section 4.3 state what the record does and does not establish about that sequence. We report it as having been falsified: one source does satisfy all four, and the claim that none does is withdrawn.

The complementary question—how much normative need goes untreated, and in whom—does not require the same conditions. It requires only that need be measured on a population frame and that treatment receipt be ascertainable. We therefore also estimate residual untreated occlusal need and its social gradient in an openly available source that permits it, and report that estimate with the definitional uncertainty it deserves.

A need-gated register is informative about treatment allocation within the eligible population: which need band receives what, and how that changes over time. That is genuine and useful information. What it cannot support is an inference about whether the eligibility threshold sits in the clinically appropriate place. A national-scale study using Norwegian population register data concluded that need-based allocation is achieved under need-criterion reimbursement, finding treatment concentrated in higher need bands [5]. We engage that finding directly rather than assume it away. It is entirely consistent with our argument: concentration of treatment across need bands among recipients is precisely the quantity a need-gated register can report, and precisely the quantity that carries no information about whether the rule is set at the right place. A register in which everyone qualifies will show excellent need-based targeting under any threshold whatsoever.

## 2. Materials and Methods

### 2.1. Registration, Amendments, and the Status of Every Analysis Reported Here

Estimands, model form, priors, decision thresholds, and stop conditions were registered before analysis, together with a disclosure of which quantities had already been observed at the time of registration (Supplementary File S2). Those quantities are named rather than merely acknowledged: the England and Wales below-threshold starts, the Norwegian recipient shares by need band, and the German KIG grade distribution had all been inspected during feasibility scoping before the registration was deposited. No NHANES III quantity had been computed. The registration is deposited under the concept DOI 10.5281/zenodo.21725476, which always resolves to the current version including all amendments. Two amendments have been registered. Amendment A-01, filed during the original study before any untreated occlusal-need result had been computed, covers the need cut-points (Section 2.6) and the appliance-status resolution (Section 2.7). Amendment A-02 declares every reviewer-requested estimation below as post hoc in advance of its being run, corrects the registration-status misstatements identified by the audit in Section 4.3, and records the falsification in Section 3.1. One exception is stated rather than glossed: the documented search of Section 2.4 was executed before A-02 was filed, under a written waiver recorded in the project’s gate file on the ground that a search and a source assessment compute no estimate. That search is what falsified the central claim, and A-02 was filed the following day carrying the result. Every analysis that produces a number about need, treatment, or the social gradient was declared before it ran.

Every analysis added in this revision is reviewer-requested and post hoc. This is stated in A-02, in each caption, and in the machine-readable claim map (Supplementary Files S3 and S7), which carries a mandatory registration status on every number in this manuscript. Of the 29 quantity families in the submitted version, the audit reported in Section 4.3 classified 7 analyses as having no registration cover.

The record does not establish that the four identification conditions were fixed before the study’s first sources were inspected. The repository’s earliest artefact is dated 27 July 2026, the conditions first appear that same day, and the registered protocol dates a feasibility inspection of three of the four original sources to 26 July 2026. We state this rather than assert the more favourable sequence.

The registration, amendments and statuses of each analysis are presented in Table 1. The study was restricted by decision to sources that are openly available without registration, application or licence. The restriction is a decision, not a discovery, and Section 4.4 states what it costs.

### 2.2. Definitions

Four distinct quantities are kept separate throughout, because conflating them is the error this paper is about.

- **Normative need**—a clinical judgement, made by an examiner against a stated criterion, that a condition warranting treatment is present. It is the quantity measured here.
- **Clinical treatment indication**—the judgement that a specific treatment is warranted for a specific individual, which incorporates growth stage, prognosis and patient factors that a population survey does not record.
- **Eligibility**—administrative qualification for reimbursement under a payer’s rule. Eligibility is a function of a need score and the threshold applied to it; the threshold is a policy instrument.
- **Treatment acceptance**—whether an eligible individual, offered treatment, takes it up. Non-acceptance and non-eligibility are indistinguishable in a claims record.

Unmet need here means normative need present with no treatment received and no appliance at examination. It does not distinguish non-referral from non-acceptance, and no claim about which predominates is made.

Over-treatment is defined in Section 1 and is a population-level prevalence, not a share of recipients.

Untreated occlusal need denotes an occlusal trait at or above the severity threshold specified for that trait, in a child with no reported history of orthodontic treatment. The term is descriptive of what the survey recorded. It does not assert that treatment was individually indicated, since individual indication incorporates growth stage, prognosis, and patient-specific factors that a population survey does not capture.

### 2.3. The Identification Conditions

We pre-specified four conditions. Population-level over-treatment is identified from a data source if and only if all four hold:

- **I1**—need is measured before treatment, on the same individuals;
- **I2**—the sampling frame includes individuals who received treatment and those who did not;
- **I3**—need measurement is not conditioned on treatment eligibility or reimbursement;
- **I4**—treatment receipt is observed irrespective of payer, including private and direct-to-consumer care.

The falsification rule was registered before any assessment: if any assessed source satisfied all four, the central claim fails and is reported as failed. The rule is implemented as an executed test in the analysis code rather than as a narrative assertion (Supplementary File S1).

### 2.4. Source Identification: The Documented Search

The submitted version of this paper assessed four country-unit sources chosen by convenience, which all three reviewers identified as insufficient support for a universal negative. That assessment is superseded by a documented search, pre-specified in a protocol dated before the first query and reported in full as Supplementary File S8.

Eight source frames were fixed in advance: OECD national statistical office catalogues; health-ministry open-data portals; the WHO Global Health Observatory; the Global Oral Health Data Portal; national dental survey series; open government data portals; PubMed; and a bibliographic frame. Four amendments to the protocol were registered during execution and are reported with their reasons, including the withdrawal of Scopus—a search behind a subscription API is reproducible only for subscribers, which is the same defect this paper identifies in need-gated records—and its replacement by keyless OpenAlex and Europe PMC. Frames that could not be searched are declared rather than omitted.

A total of 112 requests were logged with endpoint, verbatim query, timestamp, HTTP status, result count, and the universe searched. Screening applied a fixed and dated decision rule before any record was seen, and was executed by a large language model. It was a single-screener, which is a limitation. A blind re-screen of a random 10% sample was then performed by the author, working from the same decision rule and blinded to the first pass. Of 1412 records drawn, 1355 yielded comparable decisions, and 33 of those disagreed, a rate of 2.44%. That figure is an agreement between the model screener and a human screener applying the same rule. It is not an agreement between two independent human screeners, and it does not establish that the rule itself classifies records correctly, since a record that both screeners would misclassify under a poorly specified rule would not appear as a disagreement.

Candidate sources named by included records were then assessed against I1–I4 using each custodian’s own documentation, not a secondary description of it. Where a verdict turned on what a file actually contains, the file was retrieved and checked.

The 117 distinct sources named by included records were reduced to the 26 assessed against I1–I4 by three sequential criteria, applied in order and recorded per source in File S8: sources below country-unit scale were excluded; duplicate naming of the same custodian data product under different titles were merged; and sources for which neither an orthodontic, occlusal or malocclusion measure nor an orthodontic treatment-receipt variable could be located in the custodian’s own documentation were excluded, since at least one of those two elements is necessary for any of the four conditions to be assessable. A source excluded at this stage is not a source that failed the conditions. It is one to which the conditions do not apply.

### 2.5. Untreated Occlusal Need: Measures and Sample

The analysis of untreated occlusal need is cross-sectional: it uses a single examination wave of a nationally representative household survey, no individual is observed more than once, and no before-and-after comparison is available within this source. NHANES III (1988–94) records occlusal characteristics. The survey’s own dental examination manual states that there is no summary treatment-priority index number, and we constructed none. Analyses were restricted to children aged 8–17 who received the occlusal examination.

The instruments used across sources—the IOTN dental health component, the Norwegian reimbursement bands, the German need grades and the NHANES III (1988–94) occlusal battery—are not commensurable, and the registered plan forbids any crosswalk between them. Published bidirectional conversions between occlusal indices do exist [6], and we deliberately did not use one: importing an entitlement-derived index into a survey instrument would reintroduce precisely the eligibility conditioning that condition I3 is meant to exclude. No estimate is pooled across country-units anywhere in this study.

Occlusal variables use distinct sentinel codes for “could not be assessed”; these were mapped to typed missing-reason fields and never entered a numeric variable (Supplementary Figure S2). The poverty income ratio uses a separate blank-but-applicable sentinel, which was likewise excluded rather than read as a value. Column positions were parsed from each data file’s own layout specification, because the same variable occupies different columns in different files of this survey.

### 2.6. Need Definition, Its Registered Amendment, and the Sensitivity Composites

The registration did not specify an operational need threshold for the occlusal battery, and the threshold file incorrectly mapped an entitlement-based index to this estimand, although the registered analysis plan forbids crosswalks between instruments. This defect was identified during extraction, before any unmet-need value had been computed, and corrected by registered amendment A-01.

Cut-points were taken from the published analysis of these same survey variables and [7], the companion paper describing the survey’s oral health component [8], and they are therefore the instrument’s own published severity categories rather than a foreign index mapped onto it. This is a deliberate choice, not merely a convenient one: any index whose bands were calibrated against an entitlement rule would carry that rule’s threshold into the need measure and reintroduce the conditioning that I3 excludes. The cost is that the bands are those of a single national survey series and have not been validated against treatment outcome. These cut-points were established as descriptive severity categories in a prevalence analysis of these same survey variables. They have not been validated against treatment decisions, treatment outcomes, or any external criterion of clinical indication, and no such validation exists for this instrument. Where evidence of clinical consequence exists for an individual component, it is cited; that is evidence about one trait and not validation of the composite.

Need was defined as any of: overjet at or above 6 mm; overbite at or above 6 mm; open bite present; posterior crossbite present; and maxillary diastema at or above 2 mm. The 6 mm overjet cut-point has external clinical support: overjet of this magnitude is associated with traumatic dental injury [9].

Incisor irregularity was excluded from the primary composite. The index is defined in the source the survey manual itself cites [10], but that source publishes no severity bands, and the one candidate cut-point in the published survey analysis is ambiguously worded. Rather than guess, irregularity was moved to a sensitivity composite (SC-1), registered in amendment A-01. Its prevalence is now reported directly (Section 3.4).

Three sensitivity composites are reported, and their registration status differs, which the submitted version did not make clear:

- SC-1 adds anterior irregularity. Registered in amendment A-01.
- SC-2 removes the diastema criterion, on the ground that midline diastema in the mixed dentition is frequently transient and self-corrects on canine eruption [11]. SC-2 was not pre-registered. It was added after the component distribution was observed and before any unmet-need value was computed; the submitted manuscript described it as pre-specified in several places, and those statements are corrected here and in amendment A-02.
- SC-3 removes diastema and adds irregularity, so that the two corrections—one that should lower measured need and one that should raise it—are applied together. SC-3 is a new, reviewer-requested, post hoc analysis, declared in advance in amendment A-02.

The submitted version described the primary composite as a lower bound on need by construction, because it omits crowding. That claim is withdrawn. SC-3 tests it directly, and Section 3.4 reports the result.

### 2.7. Appliance Status and the Treatment-Receipt Superset

The registration required an appliance-at-examination field on every record. NHANES III (1988–94) carries no such field, and the examination manual directs that banded or bracketed teeth are examined in the usual manner, so a child in active treatment is measured mid-treatment without a flag. The registered stop condition for absent appliance coding therefore fired.

It was resolved by the observation that anyone wearing an appliance at examination has necessarily answered yes to having ever received orthodontic treatment, so that the never-treated group is a subset of those with neither prior treatment nor a current appliance. The appliance clause is thus redundant rather than missing. This resolution was registered in amendment A-01 before any unmet-need value was computed.

The residual risk is a report error in treatment receipt, which is self- or proxy-reported. We searched for a published validation of this item against clinical or claims records and found none; the corpus of 14,139 records assembled for the search in Section 2.4 contains no such study. Rather than cite a proxy, Section 2.10 describes a quantitative bias analysis over a stated plausibility range, and Section 3.7 reports it.

### 2.8. Statistical Model

The registered model is a hierarchical logistic regression with the poverty income ratio, age, and sex as covariates and a random effect for the pseudo-stratum by pseudo-primary-sampling-unit combination. Priors were declared before fitting: Normal (0, 1.5) for the intercept, Normal (0, 1) for each coefficient on the logit scale, and half-Normal (1) for the random-effect scale (Supplementary Figure S3). The survey examination weight enters as a pseudo-likelihood weight normalised to mean one so that the posterior reflects the achieved sample size rather than the population total; this is the weighted pseudo-likelihood construction of Savitsky and Toth [12], in the sense discussed by Gelman [13].

Convergence criteria were registered as non-negotiable: R-hat below 1.01, bulk effective sample size above 400 per parameter, and zero post-adaptation divergent transitions. A model that fails any of them is reported as failed and is not returned, reparameterised, or reduced to make it pass. A total of 14 models were fitted for this revision, and none failed. Missing covariate data were left missing; no outcome was imputed, and the missingness rate is reported.

For the social gradient, individuals were ranked by weighted fractional rank of the poverty income ratio and the model refitted with that rank as the exposure. The risk difference and relative index of inequality were computed by population standardisation: for each posterior draw, every individual was set to the most disadvantaged rank and then to the most advantaged rank, retaining their own age, sex and cluster effect, and the weighted mean predicted risk was taken. The exposure scale and the logit coefficients reported on it were not registered; this is one of the omissions Section 4.3 discloses.

The decomposition into a need limb and an access limb is reported throughout as two separately fitted models of two different outcomes. The limbs are not additive components of the total, and no arithmetic is performed on them. The decomposition was not pre-registered.

A contamination diagnostic was registered in advance: the model is refitted restricted to never-treated individuals, and if the deprivation coefficient shifts by more than 0.2 on the logit scale, the gradient is reported as contamination-sensitive, and the restricted fit becomes primary.

### 2.9. Design-Based Comparison

Reviewers asked whether a pseudo-likelihood with normalised weights adequately represents a stratified multistage design. We estimated the same quantities using a design-based approach and reported both. The R survey package version. 4.5 was used with Taylor-series linearisation on the survey’s own stratum, primary sampling unit, and examination weight variables, over 9 quantities. NHANES III (1988–94) ships 52 replicate weights, which NCHS documents as Fay’s method with K = 0.3 and the variance estimator v(θ) = 1/[G(1−K)^2^] × Σ(θ_j_ − θ)^2^, summing deviations about the full-sample estimate. These were used as an independent cross-check under that specification, verified against a hand-computed variance before use. Taylor linearisation is reported as primary because it uses the survey’s own stratum and primary sampling unit variables with a single documented estimator and no replication-scheme parameter to be chosen, leaving one fewer specification decision between the data and the reported interval.

All computation was carried out in Python 3.12.3 (Python Software Foundation, Wilmington, DE, USA) with pandas 3.0.5, NumPy 2.5.1, Polars 1.43.2, statsmodels 0.14.6 and Matplotlib 3.11.1, and in R 4.3.3 (R Foundation for Statistical Computing, Vienna, Austria) with survey 4.5, ggplot2 4.0.2, ggdist 3.3.3, patchwork 1.3.2, ragg 1.5.0, svglite 2.2.2 and systemfonts 1.3.1; models were fitted with CmdStan 2.39 through CmdStanPy 1.3.0. File S6 is the complete inventory.

### 2.10. Reviewer-Requested Sensitivity and Bias Analyses

Five further analyses were added at review, all declared post hoc in amendment A-02 before being run.

#### 2.10.1. Age-Band Interaction

The objection that diastema and other components are developmental predicts that the gradient should differ by age. An age band by income-rank interaction was fitted, and the within-band gradients are reported.

#### 2.10.2. Composite Refits

The full gradient analysis was repeated under SC-2 and SC-3, so that the effect of the composite choice on the equity finding can be read directly rather than inferred.

#### 2.10.3. Crowding Prevalence, and Every Component by Age Band

Summed anterior irregularity at or above 6 mm was tabulated overall and by age band, as the quantity excluded from the primary composite. Every other component and every composite was tabulated the same way (Table S1), because the developmental objection to the diastema criterion cannot be assessed from an all-ages share.

#### 2.10.4. Who Is Excluded from the Model

Children excluded for a missing poverty-income ratio were compared with those analysed across demographic, design, need-component and treatment-receipt variables using standardised mean differences, with 0.1 pre-committed as the threshold above which imbalance is reported as material. This replaces the submitted version’s crude comparison of untreated occlusal need alone, which Reviewer 1 correctly judged insufficient. The extraction was extended for the additional demographic variables, with a regression check confirming that every previously extracted value was byte-identical.

#### 2.10.5. Report Error

A quantitative bias analysis over report sensitivity was conducted from 0.80 to 1.00, and over differential under-reporting between the most and least disadvantaged up to a ratio of 2.0. The range is a plausibility range, not an empirically estimated one, because no validation source was found; it is reported as showing what follows if sensitivity lies in that range.

The interval reported at each point of that grid is not a posterior from a model refitted on the corrected outcome, and we state the construction rather than let the notation imply one. The model was not refitted at each of the grid’s points. The gradient at each point is a weighted score-based slope—a single Newton step from the null—and its bounds are obtained by rescaling the persisted posterior draws of the rank coefficient to that slope. The draws come from the SC-2 fit, while the outcome being corrected is built on the registered primary composite, so the bounds inherit the dispersion of a related but not identical model. They should be read as an indication of whether the gradient’s sign is stable under the correction, which is the question Section 3.7 asks of them, and not as credible intervals for the corrected quantity.

Treatment receipt is self- or proxy-reported, and no validation study for this item was found. So, the bias analysis reports what follows over a stated plausibility range (Figure S4).

### 2.11. Use of Generative Artificial Intelligence

We used a large language model (Claude Opus 5; Anthropic, San Francisco, CA, USA) as an assistant for tasks named rather than described in general terms: authoring and reviewing analysis code; executing the documented source search and the title and abstract screening described in Section 2.4; reconciling reported values against their source artefacts; and auditing the registration status of every reported quantity (Section 4.3). No statistical estimate was generated by the model. Every estimate was produced by executable code operating on archived, check-summed source files. Screening ran under a decision rule fixed before any record was seen, and its error rate was measured by a blind re-screen of a random 10% sample performed by the author rather than assumed. The author reviewed and verified every reported value against its provenance record and takes full responsibility for the content, the analyses, and the conclusions. The fuller statement is given under Use of Artificial Intelligence in the back matter.

## 3. Results

### 3.1. The Falsification Test Fired: One Open Source Satisfies All Four Conditions

**Figure 1:**
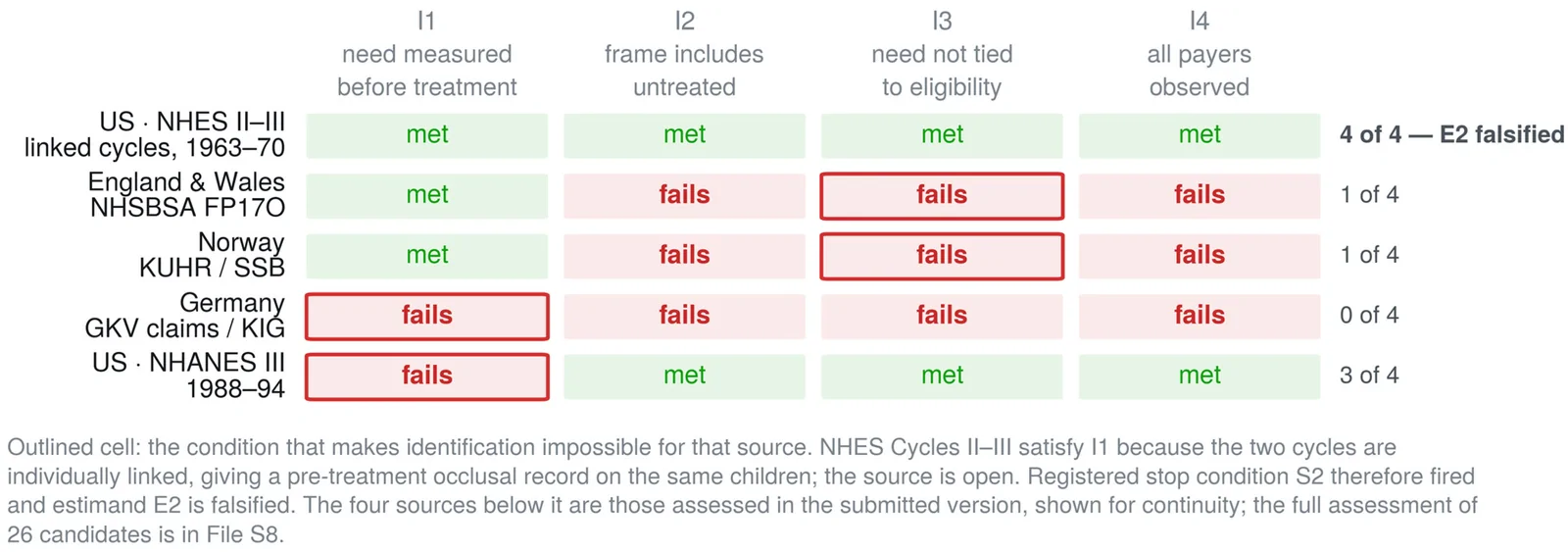
One source satisfies all four conditions: the falsification test fired.

The NHES assessment is reviewer-requested and post hoc, declared in amendment A-02, while the assessments of the four original sources were pre-registered. Of 26 sources assessed against the identification conditions, 25 fail. One satisfies all four, so the registered stop condition fired, and estimand E2 is falsified (Table 2, Figure 1).

Figure 1 is reproduced here in revised form. In the submitted version, its title asserted that no source satisfies all four conditions and that the falsification test did not fire. Both statements are now false, and the figure has been rebuilt rather than recaptioned.

The source is the US National Health Examination Survey, Cycles II (1963–65, ages 6–11) [14], and III (1966–70, ages 12–17) [15], conducted by the National Center for Health Statistics. It satisfies I1—the condition that cannot normally be repaired retrospectively—because it is two linked cycles rather than one cross-section. NCHS distributes a person-level Cycle II linkage key in the open public-use release, so for part of the Cycle III adolescent sample a pre-adolescent occlusal record exists on the same individual, measured years before any adolescent treatment.

The finding was verified against the data files themselves rather than against documentation alone. The linkage key is populated exactly as the codebook states; all 2177 individuals join to Cycle II sample sequence numbers, and Cycle II carries both a treatment.

The priority index-derived malocclusion field and an orthodontic appliance field each match their documented distribution. Excluding those already in appliance therapy at Cycle II leaves 2075 individuals for whom I1 holds cleanly. The access tier is open: both tapes and both codebooks were retrieved anonymously, with no account and no application. A licence-gated source would merely have narrowed the claim to open data, which is the narrowing the reviewers asked for; this does not.

Two limitations of the finding are recorded rather than left for a reader to discover. The linked subsample is not age-representative of the Cycle III frame, and no quantity has been estimated from it here. Establishing that a source satisfies I1–I4 completes the identification assessment; analysing it is a separate study, and this paper does not report an over-treatment estimate.

What this withdraws: The claim that no currently available open-data source identifies orthodontic over-treatment does not survive as a universal negative, and every statement of it has been removed from this paper. What it does not withdraw: the identification conditions themselves, the two structural failure families below, the Norwegian threshold-movement evidence, and the NHANES III (1988–94) unmet-need results, which concern a different half of the study.

### 3.2. How the Other Candidates Fail, and Why the Mechanism Survives

The 25 sources that do not meet all four conditions fail in the two families the framework distinguishes, which the framework accounts for, which is the part of the argument the search supports rather than undermines.

**Figure 2:**
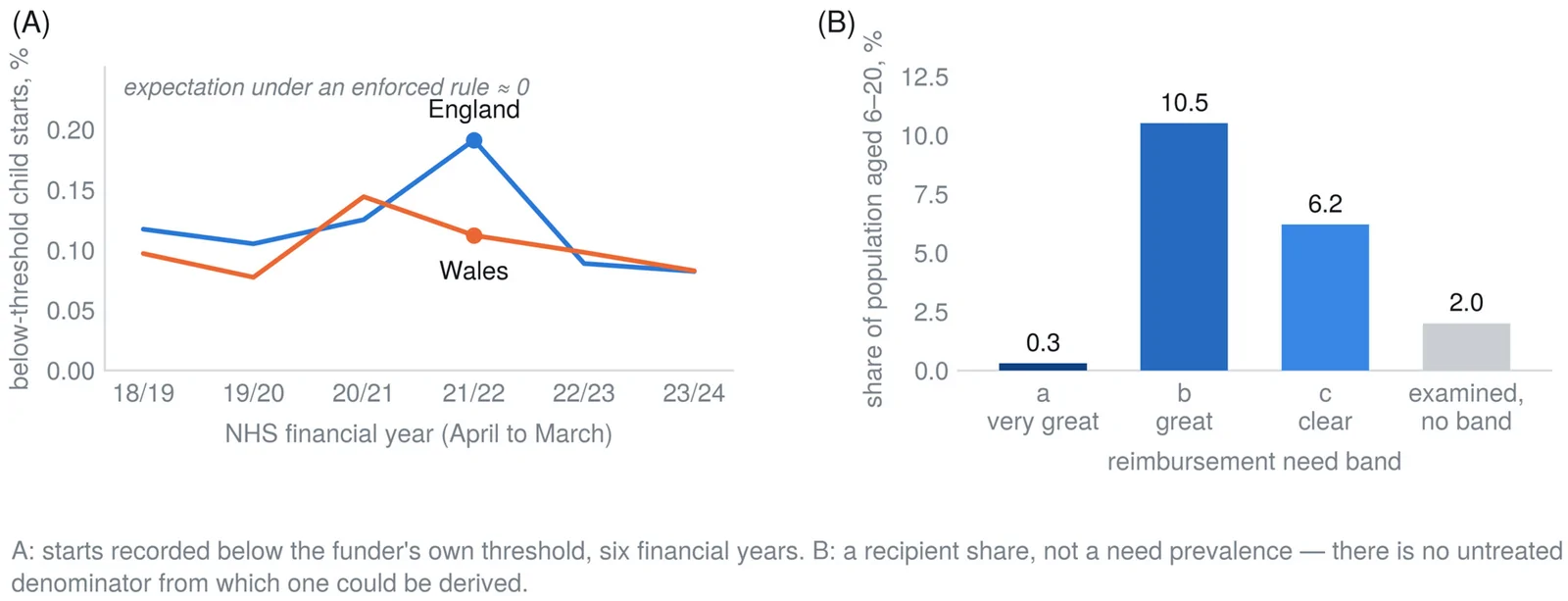
What a need-gated record contains is the eligibility rule, not the need distribution; (**A**) England and Wales, NHS orthodontic starts; (**B**) Norway, reimbursement recipients by band, 2022.

Administrative sources fail I3 by construction. A need score is recorded because it determines entitlement, so the record contains the need distribution of the eligible. One clarification a reader needs before the English figures can be read: the Index of Orthodontic Treatment Need has two components—a dental health component scoring occlusal traits, and an aesthetic component scoring appearance against a photographic scale—and NHS eligibility turns on the dental health component, with a defined additional route through the aesthetic component. Every IOTN figure in this paper is the dental health component. In England and Wales, the proportion of children whose starts were recorded below the funder’s own eligibility threshold ranged from 0.08% to 0.19% across six financial years and two nations (Figure 2A). The expectation of that quantity under an enforced rule is approximately zero, which is what makes it uninformative about threshold placement: it measures rule enforcement, not clinical judgement.

Population surveys fail I1. Occlusion is measured at examination, so for anyone already treated, the recorded occlusion is post-treatment. A treated individual with no current need therefore identifies treatment success, not unnecessary treatment. NHANES III (1988–94) meets I2, I3, and I4 and fails only I1, as does the Canadian Health Measures Survey; the greatest number met by any single source other than the falsifying one was three of four.

Because the two failure families are independent, repairing either alone would not identify over-treatment. An administrative record with a population frame would still record need as a function of eligibility; a single-wave survey with all-payer treatment ascertainment would still measure occlusion after treatment. What the falsifying source shows is that the third route—two linked waves of the same survey—closes I1 without touching either family, and that it has existed in the public domain since the 1960s.

### 3.3. The Censoring Is a Designed Response, Not an Accident

Norway documents the mechanism prospectively and from inside the system. Before 2020, the health ministry concluded that a share of reimbursements was being used to correct very minor bite deviations and that the treated share exceeded that of comparable countries. The evidential route matters: the conclusion was reached comparatively, against other countries, and not from the domestic records, because everyone in those records qualified. The response was to tighten the eligibility criteria from January 2020, after which the records again show almost everyone qualifying, now under a stricter rule. In the most recent reported year, reimbursement recipients comprised 19.0% of the population aged 6–20, distributed across bands from 0.3% in the highest-need band to 10.5% and 6.2% in the two lower bands (Figure 2B).

Germany shows the same mechanism acting on the evidence base rather than on the claims record: children in the two lowest need grades were excluded from the ministry’s own commissioned evidence review because they do not qualify for reimbursement.

The general form is this. When a system recognises over-treatment, it moves the eligibility threshold; after the move, the phenomenon is unobservable in precisely the data that would be used to measure it. A system that responds to over-treatment by changing its threshold destroys the evidence of what it detected. This is a statement about need-gated records, and Section 3.1 shows it is not a statement about every possible source.

### 3.4. Need in NHANES III Is Substantial and Definition-Dependent

**Figure 3:**
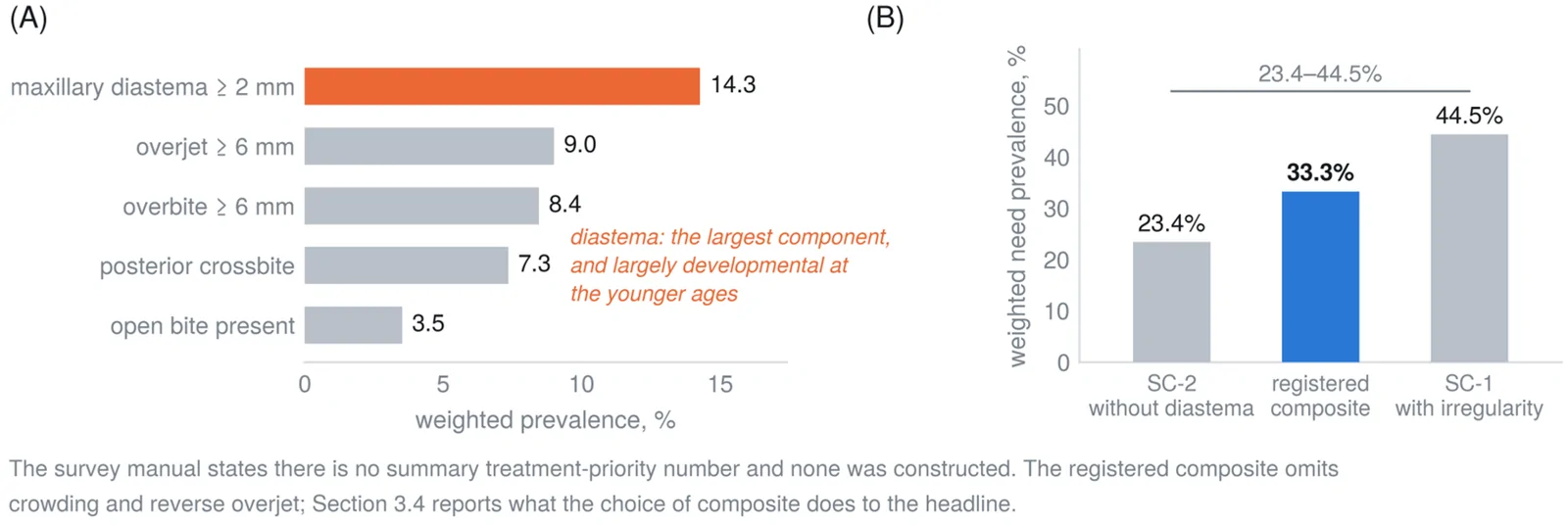
NHANES III records occlusal characteristics, not a need classification. (**A**) Components of the registered composite; (**B**) the headline depends on the definition.

Among 4593 children aged 8–17 who received the occlusal examination, weighted need prevalence under the registered composite was 33.3% (31.4% to 35.2%). Across composites, it ranged from 23.4% (SC-2) to 44.5% (SC-1), with SC-3 at 35.6% (33.6% to 37.7%). Figure 3B and Table S2 are unchanged from the submitted version and show the three composites that existed then; SC-3 and the intervals on every row are in Table S2.

That range is the most important single fact about this estimate, and we report it before the point estimate. The largest single component of the registered composite was maxillary diastema at or above 2 mm, at 14.3% weighted, which at the younger ages in this range is substantially developmental; overjet at or above 6 mm contributed 9.0%. Any headline prevalence for orthodontic need is a statement about a chosen composite at least as much as about a population.

The lower-bound claim does not hold. SC-3 applies the two corrections together—removing developmental diastema, which should lower measured need, and adding crowding, which should raise it. If the primary composite were a lower bound by construction, SC-3 would exceed it substantially. It exceeds it by +2.3 pp, and the two intervals overlap heavily. The two biases approximately cancel, so the registered composite is better described as differently specified than as conservative. The submitted version’s claim that it is a lower bound by construction is withdrawn.

The age pattern in the composite is the diastema criterion, and Table S1 shows it directly. Maxillary diastema runs 30.3% at ages 8–10, 10.1% at 11–13, and 5.1% at 14–17—a spread of 25.1 pp across the three bands, and the steepest age dependence of any component. The registered composite inherits it, falling from 46.5% to 25.3% across the same bands, a spread of 21.2 pp. Remove the diastema criterion and the age dependence largely goes with it: SC-2 runs 25.8%, 23.4% and 21.6%, a spread of 4.2 pp.

This is the clearest evidence in the paper for the reviewers’ developmental objection, and it is evidence against our own registered composite rather than for it. The pattern is what transient mixed-dentition diastema closing on canine eruption [16] would produce, and it is what a genuinely age-invariant need measure would not. We report it, and Section 4.4 states what we have and have not done in response.

Crowding itself, the component excluded from the primary composite, has a weighted prevalence of 18.8% (16.7% to 21.1%)—11.4% maxillary and 12.3% mandibular. It is steeply age-graded, from 4.2% at ages 8–10 to 22.1% at 11–13 and 27.4% at 14–17, which is consistent with late incisor crowding and is one reason a single all-ages composite is a blunt instrument.

As an external check on the extraction, the weighted proportion of children ever treated reproduced the published estimate from the same survey [6] (18.0% published for children, 20.0% for adults aged 18–50).

### 3.5. Residual Untreated Occlusal Need, and Who Is Excluded from the Model

Of children with need present (1598), 87.8% had never received orthodontic treatment. Expressed over all examined children, 29.2% had need present and had never been treated. The registered estimand text is ambiguous between these two readings; both are reported and Section 4.3 records the ambiguity rather than resolving it retrospectively.

The model was fitted to 4219 children across 98 sampling units; 8.1% were excluded for a missing poverty income ratio. All registered convergence criteria were met: maximum R-hat 1.0011, minimum bulk effective sample size 1911, and no post-adaptation divergent transitions (Supplementary Figure S6). The posterior predictive check for weighted prevalence gave a Bayesian *p*-value of 0.524 (Supplementary Figure S7). The registered contamination diagnostic did not fire: the deprivation coefficient shifted by 0.112 on the logit scale between the full and never-treated fits, within the registered trigger of 0.2 (Supplementary Figure S8). A reviewer noted that this is more than half the distance to a threshold we set ourselves, and that is the fairer way to report it than “did not fire”. The diagnostic passed; it did not pass comfortably, and Section 4.4 treats occlusal alteration by prior treatment as a live concern rather than a discharged one.

The excluded children differ from those analysed, and it matters. Comparing the 374 children excluded with the 4219 children analysed across 26 variable levels, seven exceed the pre-committed threshold of 0.1 (Table S3). The largest is race/ethnicity at 0.400; region, urbanicity, and the design stratum also exceed it.

The pattern is as informative as the count. Every exceedance is demographic or design. No need component, no composite need status, and no treatment-receipt variable exceed the threshold, and the largest clinical imbalance is 0.096. The excluded children differ in who they are, not in what was found on their examination. That is reassuring for the outcome model and not reassuring for the exposure: in a 1988–94 US survey, race/ethnicity is strongly associated with income, and income is the exposure. Section 4.4 states the consequence.

### 3.6. The Social Gradient: Total, by Composite, and by Age Band

Among 4593 children aged 8–17 who received the occlusal examination, weighted need prevalence under the registered composite was 33.3% (31.4% to 35.2%). Across composites, it ranged from 23.4% (SC-2) to 44.5% (SC-1), with SC-3 at 35.6% (33.6% to 37.7%), and this range is the most important single fact about the estimate.

**Figure 4:**
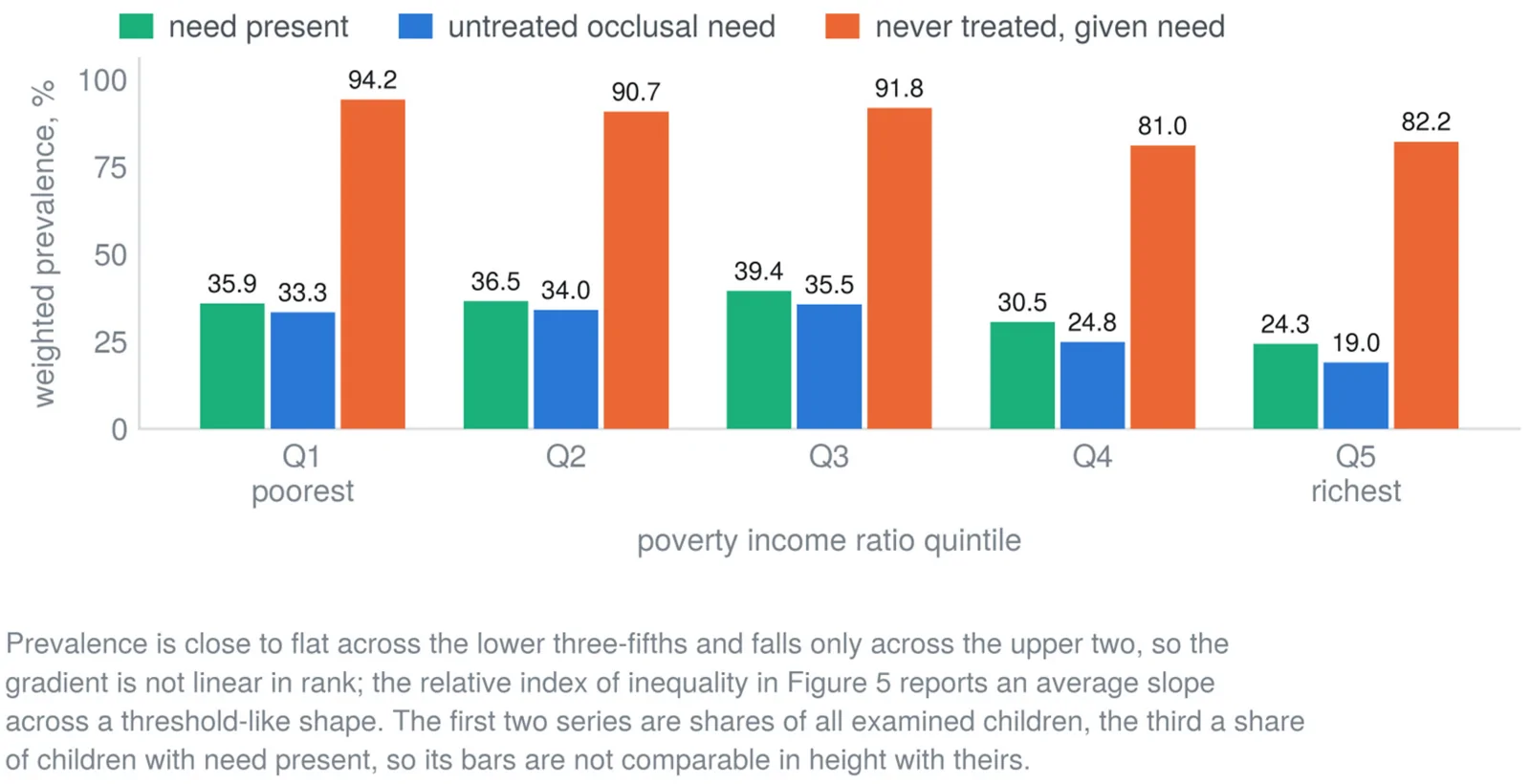
Untreated occlusal need by poverty income ratio quintile.

Untreated occlusal need was strongly socially graded. Standardised risk fell from 37.6% at the most disadvantaged rank to 21.7% at the most advantaged—a risk difference of 15.9 pp (11.1 pp to 20.6 pp) and a relative index of inequality of 1.74 (1.46 to 2.05) (Table 3, Figure 4). Following Reviewer 3, the risk difference is now reported as the primary summary and the relative index as secondary.

The gradient is not linear in rank. Prevalence is close to flat across the lower three-fifths of the income distribution and falls only across the upper two-fifths (Figure 4). The relative index of inequality assumes linearity in rank and therefore reports an average slope across a shape that is closer to a threshold; the quintile pattern is presented alongside it for that reason, and the index should be read as a summary rather than as a description of the shape.

#### 3.6.1. The Finding Does Not Depend on the Composite

Refitting under SC-2, which removes the developmentally suspect component, gives a risk difference of 12.6 pp (8.2 pp to 17.0 pp) and a relative index of 1.91 (1.52 to 2.39); under SC-3, 22.5 pp (17.6 pp to 27.1 pp) and 2.15 (1.81 to 2.53). The absolute difference is smaller under SC-2 than under the registered composite, and the relative index is larger, so the choice of composite moves the magnitude without touching the direction or the significance (Table S4).

#### 3.6.2. The Gradient Does Depend on Age, and in a Way That Supports the Developmental Objection

Within ages 8–10, the rank gradient is −0.261 (−0.655 to +0.137), and its credible interval includes zero (Supplementary Figure S9). Within ages 11–13, it is −1.373 (−1.828 to −0.912) and within 14–17, −1.042 (−1.451 to −0.626). The overall gradient is therefore driven by children aged 11 and over, and we do not claim one in the youngest band.

The access limb is located where there is no age difference. Fitted within the same bands, the chance of remaining untreated given need is near-identical across all three: −1.738, −1.950 and −1.689. Since the total gradient varies by band and the access gradient does not, the age dependence must sit in the need limb. We state that as an inference from the two fitted quantities and not as a third measurement: the need limb was not fitted by age band, and Table S4 reports the limbs for the sample as a whole.

The inference is what the developmental-component objection predicts. Table S1 shows the mechanism directly: maxillary diastema, the largest single component of the registered composite, is concentrated at the younger ages where it is most likely to be transient, so at 8–10 the composite is picking up findings that are not socially patterned. They dilute the total gradient without touching the access gradient underneath it.

#### 3.6.3. The Decomposition Is Scale-Dependent, and Its Limbs Are Not Additive

**Figure 5:**
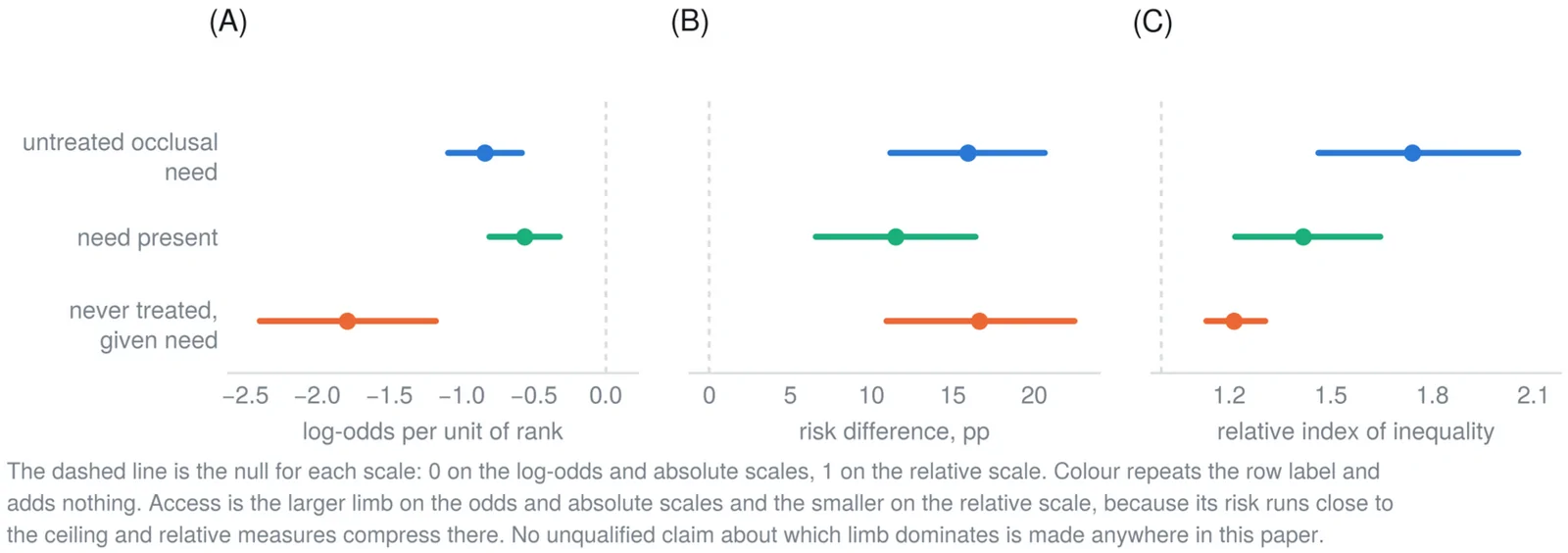
The need and access limbs order differently depending on the scale; (**A**) odds scale; (**B**) absolute scale; (**C**) relative scale.

They come from two separately fitted models of two different outcomes. On the odds scale, the access limb is the larger (−1.79 against −0.56 on the logit scale), as it is on the absolute risk difference (16.6 pp against 11.5 pp). On the relative index of inequality, the ordering reverses: 1.21 (1.13 to 1.31) for access against 1.42 (1.22 to 1.65) for need. All three scales are shown together in Figure 5.

The reversal is a ceiling effect rather than a contradiction. Risk of remaining untreated given need runs from 94.7% to 78.0%, close enough to the upper bound that relative measures compress while odds-scale measures expand. Every statement about which limb dominates must name its scale in the same breath. On absolute and odds scales, the social gradient in untreated occlusal need operates substantially through whether treatment is received rather than through how much is needed; on the relative scale it does not, and we make no unqualified claim.

### 3.7. Report Error Cannot Explain the Gradient Away

The first result inverts the usual intuition. Non-differential under-reporting cannot explain the gradient away, because it biases the association toward the null: correcting it makes the gradient stronger, not weaker. Asking at what report sensitivity the gradient disappears therefore has no answer, and the question as usually posed is the wrong one.

Only differential under-reporting threatens the finding. Varying the excess of under-reporting among the most disadvantaged from parity up to 2.0×, the rank gradient moves from −0.693 (−0.943 to −0.452) at parity, to −0.565 (−0.769 to −0.368) at 1.5×, to −0.408 (−0.554 to −0.266) at 2.0×. The interval excludes zero at every point examined—zero of the grid points include zero—so there is no tipping point within that range. These bounds are rescaled posterior draws around a score-based slope rather than posteriors from a refit, as Section 2.10 sets out; they support the statement that the gradient’s sign is stable under the correction, and no more than that.

On the level rather than the gradient, correcting non-differential under-reporting at the most pessimistic sensitivity considered moves untreated occlusal need from 29.3% to 27.7%, a change small relative to the range induced by the composite choice in Section 3.4.

### 3.8. Design-Based and Bayesian Estimation Agree

Estimating the same quantities with Taylor linearisation on the survey’s own design variables reproduces the Bayesian results (Table S5). Need prevalence is 33.31% (30.6% to 36.0%) against 33.3%; untreated occlusal need over all examined children 29.23% (26.9% to 31.6%); never treated given need 87.75% (85.1% to 90.4%); and the rank gradient -0.861 (−1.171 to −0.551) against −0.838. The largest absolute difference on any prevalence is 0.046 pp, and no quantity diverges materially. The concern that a normalised pseudo-likelihood weight might misrepresent a stratified multistage design is not borne out here.

The replicate-weight cross-check initially disagreed with Taylor linearisation by a factor of about four on the standard errors while reproducing the point estimates exactly. That shape identified the fault: a variance multiplier cannot move a point estimate, so the weights were being read correctly and only the multiplier was wrong. Two errors were found. The replicate design had been declared with a jackknife multiplier of (G − 1)/G = 0.980769 rather than the documented Fay multiplier of 1/[G(1 − K)^2^] = 0.0392465, a ratio of 5.00 on the standard-error scale; and squared deviations were being summed about the mean of the replicate estimates rather than about the full-sample estimate, which the documented formula requires. Both are corrected here, and the corrected variance was verified to six decimal places against a hand computation.

Under the documented specification, the two design-based routes agree. Point estimates are identical to within 5 × 10^−4^ across all nine quantities, and replicate standard errors run between 0.79 and 1.05 times the Taylor ones. They are not expected to agree exactly: the replicate weights carry the survey’s nonresponse and poststratification adjustments, which Taylor linearisation treats as fixed, and that generally lowers the replicate variance. The residual difference is of that kind. Table S5 reports both the corrected and the superseded standard errors, and the superseded estimates are retained in the archived record.

## 4. Discussion

### 4.1. What Survives and What Does Not

The paper’s central claim as submitted does not survive. We stated that no currently available open-data source identifies orthodontic over-treatment, registered the rule that would falsify it, ran the search the reviewers required, and the rule fired. A source exists; it is open and has been open since the 1960s. The claim is withdrawn, not qualified.

Three things survive, and they are what the paper now rests on.

The identification conditions survive as a usable instrument. They were stated in advance, they were falsifiable, and they were falsified by an assessment run against them—which is the behaviour a framework should exhibit. The conditions also classified the failure pattern consistently: of 26 sources assessed, 25 fail, and they fail on I3 if administrative and on I1 if a single-wave survey, with no unexplained third family.

The mechanism survives. Estimates of orthodontic over-treatment derived from need-gated administrative records—including below-threshold shares of the kind reported here for England and Wales—should not be read as measurements of unnecessary treatment. Their expectation under an enforced rule is approximately zero regardless of how much unnecessary treatment occurs, so they carry essentially no information about where the threshold should be placed. They may well carry information about coding behaviour, administrative compliance, or divergence between clinical and reimbursement decisions, and we no longer imply otherwise. The narrower claim is the defensible one and is the one we make.

The untreated occlusal-need results survive untouched, because they never depended on the identification claim. Untreated occlusal need in this survey population was substantial and strongly socially patterned, with the disadvantaged bearing both more need and, even when accounting for that need, considerably less treatment.

What the falsification changes for the field is the interesting part. The route that closes I1 is not a new instrument or a new linkage agreement: it is two waves of the same examination survey on the same individuals. That design existed sixty years ago and has not been repeated. Section 4.6 takes up what follows.

### 4.2. Relation to Prior Work

None of the individual components here is new. The normative/felt/expressed taxonomy of dental need is four decades old [8]. Quantification of treatment failing to meet a funder’s criteria is three decades old [2,3] and has been repeated by audit [4]. The two-sided misallocation framing is stated in the field’s flagship series [1]. Published pooled malocclusion prevalence exists and is dentition-stratified [17], and the measurement properties of occlusal indices have been studied directly, including anchor-based threshold calibration [18] and latent-trait treatments [19].

What this paper adds is the identification statement: an explicit, falsifiable enumeration of when the population-level over-treatment quantity is recoverable at all, applied to the sources that exist, together with the result of applying it. The four conditions are this paper’s proposal and not an established framework. A methodologist could reasonably propose a fifth, or argue that I2 and I3 collapse into one another in some designs. We state them plainly so that they can be argued with, and we have now demonstrated that they can be used to overturn the claim they were built to support.

I2 in particular deserves the scrutiny a reviewer gave it. It is the condition that separates the two estimands defined in Section 1: a treated-only record can support a statement about inappropriate treatment among recipients, and I2 is what a population-level prevalence additionally requires. A reader who is interested only in the first quantity does not need I2, and should not read its failure as a defect for their purpose.

The falsification bears on the conditions themselves and not only on the data landscape. A condition set that a survey fielded in the 1960s satisfies while every modern source fails is saying something about how examination surveys have changed—occlusal batteries have been dropped, and repeat examination of the same individuals is no longer done—as much as about the conditions being demanding. We record that reading rather than presenting the conditions as settled.

The Norwegian register study concluding that need-based allocation is achieved [5] deserves specific attention because it appears to contradict the framing here but does not. That study measures concentration of treatment across need bands among those who received reimbursement. That is exactly the quantity a need-gated register supports, and exactly the quantity that is uninformative about threshold placement: a register in which nearly all recipients qualify will exhibit strong need-based targeting whatever the threshold. The two results are compatible, and read together they sharpen the point—good targeting within the eligible population is not evidence about whether the eligibility boundary is correct.

The conditions were applied to sources rather than tested against them prospectively, so their consistency with the observed failure pattern is a property of how well they classify and not independent validation.

### 4.3. Registration Status of the Analyses Reported

We audited, and the audit contradicted our own declaration. The full register is File S9; the corrections are registered in amendment A-02. The audit finds seven unregistered analyses. Figure S1 is the only one declared as post hoc. Of the other six, three feed a headline claim. A further two computational settings are also unregistered; they are listed for completeness and are not analyses. Separately, one registered estimand is ambiguous between two readings that the manuscript reports side by side. It is classified as registered, not post hoc, with the ambiguity recorded rather than resolved.

Two further disclosures the audit produced:

Estimand E3’s registered text is ambiguous between a joint reading (untreated occlusal need overall among examined children, 29.2%) and a conditional one (never treated among those with need, 87.8%). Both are reported, and neither was concealed, but the choice of which is primary was recorded in a code comment rather than in a registered document. The ordinary reading of “the proportion of children with need who…” favours the conditional, which is the value the Abstract quotes.

### 4.4. Limitations

The central claim was falsified by our own search, which is the first limitation and is stated in Section 3.1 rather than here.

The untreated occlusal-need estimate rests on a single country-unit and a survey fielded between 1988 and 1994. It is not a contemporary estimate, and it is not transportable; no cross-national comparison was attempted, and none should be inferred. The open-data-only restriction is the direct cause of this narrowness, and it was a decision rather than a discovery.

No contemporary US survey carries a comparable battery. Across the three NHANES dentition codebooks for 2011–2016, there are no occurrences of any of the nine occlusal terms searched in codebooks documenting 107, 107, and 167 variables, respectively. The absence is of the measurement, not of the documents. No later NHANES cycle carries a comparable occlusal battery, so the estimates cannot be updated from the same instrument.

Need is defined by a composite whose choice moves the headline substantially, as Section 3.4 shows. We no longer describe the registered composite as a lower bound; SC-3 shows the two directions of misspecification approximately cancelling.

A reviewer recommended promoting SC-2 to the primary estimate, on the ground that midline diastema in the mixed dentition is developmental. The case is reasonable, and the full SC-2 results are reported alongside the registered ones in Table S4, where a reader can adopt them. We have not made the substitution because the primary composite is the registered one, and changing it after seeing that the substitution strengthens the relative gradient would be precisely the decision a pre-registration exists to prevent. Reporting both is the honest resolution; choosing the more favourable one after the fact is not.

Children excluded for a missing poverty-income ratio differ demographically from those analysed, on seven of 26 variable levels including race/ethnicity at 0.400. Because race/ethnicity is strongly associated with income in this population and income is the exposure, missingness cannot be treated as ignorable for the gradient. The submitted version’s defence—that crude untreated occlusal need was similar among the excluded—is measurably insufficient rather than merely unproven. The gradient estimates should be read as conditional on being in the analysed sample.

Treatment receipt is self- or proxy-reported and unvalidated. The bias analysis in Section 3.7 shows the gradient surviving differential under-reporting to 2.0×, but this is a plausibility range and not an empirical one.

The social-gradient decomposition is scale-dependent; its limbs are separately fitted models of different outcomes rather than additive components; the relative index of inequality assumes a linearity the quintile pattern does not display; and the decomposition was not pre-registered.

In children aged 8–10, absence of treatment may reflect appropriate deferral to a later developmental stage rather than failed access, because much orthodontic treatment is deliberately timed to the permanent dentition. “Untreated occlusal need” should be read with that qualification in the youngest band, which is also the band in which we do not claim a gradient (Section 3.6).

The registered contamination diagnostic passed, but by less than half the margin we allowed it. Occlusal alteration by prior treatment is therefore a live limitation of the unmet-need estimate rather than one the diagnostic has discharged.

The design is cross-sectional. No causal interpretation of the gradient is supported, and none is offered.

A single-screener design with human verification on a 10% sample is weaker than dual independent human screening.

### 4.5. Generalisability

Two generalisability questions arise here, and they have different answers.

The identification result generalises as an argument rather than as an estimate. The four conditions are a property of how a data source is constructed and not of a population, so the finding that need-gated administrative records fail I3 holds wherever a need score determines entitlement, which is the usual arrangement where orthodontic treatment is publicly funded against a threshold. The count is narrower than the argument: 25 of 26 sources failing is a property of the frame searched and the date it was searched, and it would change as sources are opened or added. The falsifying source is a US survey, and the search was restricted by decision to sources that are open without registration, application, or licence; whether a comparable linked pair exists behind an application, in the United States or elsewhere, is a question this study cannot answer.

The untreated-need estimates generalise much less far. They describe US children aged 8 to 17 examined between 1988 and 1994, and they should be read as describing that population at that time. Three things bound them. The survey is more than thirty years old, and no later NHANES cycle carries a comparable occlusal battery, so the estimates cannot be refreshed from the same instrument and are not contemporary. The instruments used elsewhere are not commensurable with it, and the registered plan forbids a crosswalk between them, so these figures do not transfer to settings that classify need with the IOTN dental health component, the Norwegian reimbursement bands or the German need grades. And the gradient estimates are conditional on being in the analysed sample, because the children excluded for a missing poverty income ratio differ from those analysed on characteristics associated with the exposure.

What is most likely to carry beyond this sample is the structure rather than the magnitude: that need measured on a population frame is substantially larger than a treated-case record implies, and that the residual is socially patterned. Both follow from the measurement position, not from the particular composite or the particular survey year. The magnitudes do not, and nothing here licences transporting them.

### 4.6. Implications

For research practice, a study proposing to measure orthodontic over-treatment should state, before collecting anything, which of the four conditions its source satisfies, and the shortfall should appear in the abstract rather than in the limitations. On present evidence, the answer for a need-gated record will be that it satisfies at most three, and the failing one will be I3.

Rather than advocating for costly new data infrastructure or novel linkage laws, this paper’s findings redirect measurement policy toward a proven, pragmatic alternative. Section 3.1 demonstrates that the identification gap can be closed using a standard epidemiological framework: repeated examinations of a population-frame cohort before and after the typical age of treatment, utilising a measurement instrument with no entitlement consequences. Because this exact framework was successfully executed by the National Health Examination Survey in the 1960s, the most actionable next step for measurement policy is to analyse currently linked cycles and replicate this design on a contemporary cohort.

The obstacles are practical and political rather than technical. Occlusal examination adds cost and examiner-training burden to a survey whose content is competitively allocated; a measure with no entitlement consequence has no administrative constituency arguing for its retention; and a second wave requires a commitment across a survey cycle boundary. None of these is an argument against the design. They are an account of why the design exists once, sixty years ago, and not since.

Future work should focus on a service policy, the finding that the income gradient in untreated occlusal need operates substantially through treatment receipt rather than through need points at access rather than at disease distribution—with the caveats, stated in Section 3.6, that this holds on absolute and odds scales and reverses on the relative one, that the limbs are not additive, that the decomposition was not pre-registered, and that the gradient is not established within the youngest age band.

## 5. Conclusions

In the need-gated administrative records from which it is usually estimated, population-level over-treatment is not identified by the sources assessed here. The reason is structural: when a health system recognises over-treatment, it moves the eligibility threshold, and after the move the phenomenon becomes difficult or impossible to identify within the same reimbursement dataset. Below-threshold shares from such data do not estimate the population prevalence of unnecessary care, and are better reported as measures of rule enforcement than as measures of clinical judgement.

Though considered impossible before, the residual untreated occlusal need, by contrast, is measurable where a population frame exists, and in the openly available source that permits the estimate it is substantial and steeply socially graded—a gradient that survives changes in composite, differential report error, and design-based re-estimation, but that is not established below age 11.

## Figures and Tables

**Table 1:** Registration, amendments, and the status of analyses.

Analysis Group	Registration Status	Where It Is Reported
The identification conditions and the four original source assessments	Pre-registered	Section 2.3, Section 3.1, Section 3.2 and Section 3.3
The need for cut-points and appliance-status resolution	Amendment A-01	Section 2.6 and Section 2.7
The documented search and all nine reviewer-requested analyses	Amendment A-02 and declared post hoc in advance	Section 2.4, Section 2.10, Section 3.1, Section 3.4, Section 3.6, Section 3.7 and Section 3.8
SC-2, both decomposition limbs, the rank-gradient logit coefficients, the quintile estimates, the excluded-child comparison and Figure S1	Post hoc and undeclared at submission	(but now declared in) A-02, Section 4.3 and File S9.

**Table 2:** Assessment of the five country-unit data sources named in the text against the four identification conditions.

Source	Record Type and Need Instrument	I1	I2	I3	I4	Met	Decisive Failure
United States, NHES Cycles II (1963–65) and III (1966–70), linked	Population examination survey, two linked cycles; treatment-priority index (Grainger)	✓	✓	✓	✓	4 of 4	— (satisfies all four)
United States, NHANES III (1988–94)	Population examination survey; occlusal battery, no summary index	✗	✓	✓	✓	3 of 4	I1, occlusion recorded at examination, post-treatment in the treated
England and Wales, NHSBSA FP17O, release FOI-02868	Publicly funded orthodontic starts (Supplementary Figure S5); IOTN dental health component	✓	✗	✗	✗	1 of 4	I3, the need score is recorded because it sets entitlement
Norway, KUHR register, reported in SSB Rapporter 2023/37	Reimbursement claims; statutory need bands a, b, c	✓	✗	✗	✗	1 of 4	I3, the bands are the reimbursement criteria
Germany, GKV claims	Statutory insurance claims; KIG grade absent from the claims record	✗	✗	✗	✗	0 of 4	I1, the KIG grade sits in the treatment and cost plan, not the claim

✓ = condition satisfied; ✗ = not satisfied. I1, need measured before treatment on the same individuals; I2, sampling frame includes treated and untreated individuals; I3, need measurement not conditioned on eligibility or reimbursement; I4, treatment receipt observed irrespective of payer. The decisive failure is the condition that cannot be repaired for that source. NHES Cycles II–III satisfy I1 because they are two linked cycles: a person-level Cycle II key in the open public-use release gives a pre-adolescent occlusal record for 2177 of the 6768 Cycle III examinees, of whom 2075 were not already in appliance therapy at Cycle II. All five sources are open-access. The full documentary basis for every verdict, and the assessment of the remaining 21 candidates, is given in Files S1 and S8. Assessment of NHES is reviewer-requested and post hoc, declared in amendment A-02; the other four verdicts are pre-registered.

**Table 3:** Social gradient in untreated orthodontic need and its exploratory decomposition, NHANES III children aged 8–17 examined in 1988–1994.

Outcome	*n*	Risk, Most Disadvantaged, %	Risk, Most Advantaged, %	Risk Difference, pp	RII
Untreated need (need present and never treated)	4219	37.6 (34.7–40.7)	21.7 (19.4–24.3)	15.9 (11.1–20.6)	1.74 (1.46–2.05)
Need limb: need present (exploratory)	4219	39.2 (36.3–42.2)	27.7 (25.1–30.4)	11.5 (6.6–16.4)	1.42 (1.22–1.65)
Access limb: never treated among those with need (exploratory)	1473	94.7 (92.6–96.3)	78.0 (73.3–82.5)	16.6 (10.9–22.5)	1.21 (1.13–1.31)

Values in parentheses are 95% credible intervals; pp, percentage points; RII, relative index of inequality. Risks are population-standardised posterior model-based contrasts between the most disadvantaged and the most advantaged weighted fractional rank of the poverty income ratio; they are not observed proportions. The risk difference is the primary summary measure; the RII assumes linearity in rank, which the quintile pattern in Figure 4 does not display, and is reported as secondary. The three rows are separately fitted models of different outcomes: the need and access limbs are not additive components of the total and are not expected to sum to it on any scale. Neither limb was pre-registered; both are reported as exploratory (Section 4.3, File S9). The access-limb sample is the subset of the modelled sample with need present.

## Data Availability

All primary sources are publicly available without registration. Each retrieved file is recorded with its source address, retrieval timestamp, byte size, and SHA-256 checksum. Analysis code, the pre-registered protocol, both registered amendments, and a claim map linking every number in this manuscript to the artefact and source file it derives from are archived under the concept DOI 10.5281/zenodo.21725476, which resolves to the current version including amendment A-02. No restricted or licence-gated data were used.

## Supplementary Materials

The following supporting information can be downloaded at: https://www.mdpi.com/article/10.3390/healthcare14203395/s1, Figure S1: need and untreated occlusal need by age and sex, post hoc descriptive. Figure S2: completeness of the occlusal battery, showing how “could not be assessed” codes were handled. Figure S3: prior against posterior for each regression parameter. Figure S4: the report-error bias analysis, post hoc. Figure S5: the full recorded need composition of publicly funded starts in England and Wales. Figure S6: posterior intervals and convergence diagnostics for every model parameter. Figure S7: posterior predictive check for the unmet-need model. Figure S8: the registered contamination diagnostic. Figure S9: the rank gradient by age band, post hoc. Table S1: every need component and all four composites by age band, post hoc. Table S2: the need components and all four composites, with confidence intervals and a registration status on every row. Table S3: design-based versus Bayesian estimation, post hoc. Table S4: the full gradient analysis under SC-2 and SC-3, post hoc. Table S5: included against excluded children, standardised mean differences, post hoc. File S1: the full identification statement, with the per-source evidence behind every verdict. File S2: the registered protocol [20]. File S3: registered amendment A-01. File S4: a STROBE compliance mapping. File S5: a GATHER compliance mapping. File S6: an inventory of all software and tools with versions. File S7: the claim map, linking every number in this manuscript to the artefact, the code, the check-summed source file, and the registration status it carries. File S8: the documented data-source search—protocol, amendments, request log, screening log, PRISMA counts, measured screener reliability, and the falsification finding with its verification. File S9: the registration-status register for the submitted version, with the audit method and the dissent on record. Reporting follows STROBE for observational studies and GATHER for health-estimate reporting; the compliance mappings for both are supplied as Files S4 and S5. Files S4 and S5 are compliance mappings keyed to the official item numbering rather than reproductions of the copyrighted checklist instruments.

## Abbreviations

The following abbreviations are used in this manuscript:

Abbreviation	Definition
CrI	Credible interval
DHC	Dental health component (of the IOTN)
GATHER	Guidelines for Accurate and Transparent Health Estimates Reporting
IOTN	Index of Orthodontic Treatment Need
KIG	Kieferorthopädische Indikationsgruppen (German orthodontic indication groups)
KUHR	Kontroll og utbetaling av helserefusjon (Norwegian health reimbursement register)
NHANES III	Third National Health and Nutrition Examination Survey (1988–1994)
NHES	National Health Examination Survey (United States, Cycles II and III, 1963–1970)
NHSBSA	NHS Business Services Authority
PIR	Poverty income ratio
pp	Percentage points
PSU	Primary sampling unit
RII	Relative index of inequality
SC-1	Sensitivity composite adding anterior irregularity; registered in amendment A-01
SC-2	Sensitivity composite removing diastema; not pre-registered (see Section 4.3)
SC-3	Sensitivity composite removing diastema and adding irregularity; reviewer-requested, post hoc
STROBE	Strengthening the Reporting of Observational Studies in Epidemiology
TPI	Treatment Priority Index
