# Supplementary material for: When Can Orthodontic Over-Treatment Be Measured? A Pre-Registered Identification Assessment, a Falsified Claim, and a Cross-Sectional Analysis of Untreated Occlusal Need in NHANES III
Source: Healthcare (Basel). 2026 Oct 12;14(20):3395. doi: 10.3390/healthcare14203395 (PMC13704488; doi:10.3390/healthcare14203395)
Supplement: Supplementary file 1 [file healthcare-14-03395-s001.zip › File_S1_Identification_statement.pdf]

File S1 - formal identification statement (revised)

Supersedes the submitted version, whose verdict the search overturned.

File S1 - Formal identification statement (revised)

This file supersedes the version submitted with the original manuscript. That version concluded that no source satisfies all four conditions and that estimand E2 was not falsified. The reviewer-mandated search overturned that verdict, and this statement is regenerated from the assessment rather than edited, so the conclusion cannot drift from the table it rests on.

Estimand E2 (registered protocol §3). The four conditions are this paper's proposal, not an established framework. The registration record does **not** establish that they were fixed before the study's first sources were inspected: the earliest artifact is dated 2026-07-27, the conditions first appear that same day, and the protocol dates a feasibility inspection of three of the four original sources to 2026-07-26. The submitted version asserted the more favourable sequence; amendment A-02 corrects it and so does this file.

The four conditions

- **I1** - Need measured BEFORE treatment, on the same individuals
- **I2** - Frame includes treated AND untreated individuals
- **I3** - Need measurement NOT conditioned on eligibility or reimbursement
- **I4** - Treatment receipt observed IRRESPECTIVE OF PAYER (incl. private and DTC)

Over-treatment is identified from a data source if and only if all four hold. Registered stop condition S2: if any source satisfies all four, E2 is falsified and the central claim is reported as failed.

Verdict

1 source satisfies all four conditions. Stop condition S2 has fired, and estimand E2 is FALSIFIED.

United States - NHES Cycles II (1963-65) and III (1966-70), linked
Conditions met: 4 of 4. Access tier: open.

Condition	Verdict	Basis
**I1**	met	2,177 youths examined in both cycles; the occlusal assessment at ages 6-11 precedes the adolescent treatment window
**I2**	met	appliance-wearers are retained in the examined sample
**I3**	met	TPI is an epidemiological research index; the US had no national orthodontic benefit in 1963-70 for it to encode
**I4**	met	lifetime parent report, no payer qualifier

Verdicts as recorded in amendment A-02 section 2b (DOI 10.5281/zenodo.22768187). Verified against the public-use tapes, not documentation alone.

Limitations of this finding, recorded rather than left to be found. The linked subsample is not age-representative of the Cycle III frame. No quantity has been estimated from this source here: establishing that it satisfies I1–I4 completes the identification assessment, and analysing it is a separate study.

The sources assessed in the submitted version

Retained for continuity. They fail as the framework predicts: population surveys on I1, administrative records on I3, which is the part of the argument that survives.

Source	I1	I2	I3	I4	Met	Decisive failure
England & Wales — NHSBSA FP17O / FOI-02868	met	fails	fails	fails	1 of 4	I3
Norway — KUHR / SSB Rapporter 2023/37	met	fails	fails	fails	1 of 4	I3
Germany — GKV claims / KIG	fails	fails	fails	fails	0 of 4	I1
United States — NHANES III (1988-94)	fails	met	met	met	3 of 4	I1

The full search that produced the falsifying source, its protocol, its logs, and its measured screener reliability are in **File S8**. That file also records, in its §6, which of its summary counts are reproducible from deposited artifacts and which are transcribed from the filed amendment.
