# Supplementary material for: When Can Orthodontic Over-Treatment Be Measured? A Pre-Registered Identification Assessment, a Falsified Claim, and a Cross-Sectional Analysis of Untreated Occlusal Need in NHANES III
Source: Healthcare (Basel). 2026 Oct 12;14(20):3395. doi: 10.3390/healthcare14203395 (PMC13704488; doi:10.3390/healthcare14203395)
Supplement: Supplementary file 1 [file healthcare-14-03395-s001.zip › File_S2_Registered_protocol.pdf]

Supplementary File S2. Registered protocol

Registered before analysis. Amendment A-01 is reproduced as S6.

GOCAA Design C - pre-registration

Study. Need-gated reimbursement makes orthodontic over-treatment unobservable: threshold censoring and residual unmet need across five country-units.

Design. Cross-national descriptive and analytic study of routinely collected administrative data and national oral-health survey microdata. **Not a systematic review**; PROSPERO does not apply.

Registration route: Zenodo (deposition 21725477, reserved DOI 10.5281/zenodo.21725477). Zenodo provides the immutable timestamped DOI that a preregistration requires. It is not, however, indexed as a registry; a parallel OSF Registries entry was recommended and is recorded as an open advisory in `state/GATES/G1.REQUEST.yaml`.

Status. DRAFT, awaiting human submission and countersignature (gate G1, task T-02-002).

No outcome analysis may run until `state/GATES/G1.yaml` is signed.

Version. 1.1 · drafted 2026-07-27, revised 2026-08-01 · snapshot frozen at `00_snapshot/MANIFEST.jsonl` (21 files, 308,626,593 bytes, all HTTP 200).

Changes in 1.1: open-data-only constraint applied (§2.0); CDHS 2013 excluded and stop condition S4 fired by decision; E3/E4 narrowed to NHANES III alone; the amendment window of §2.0 was exercised on 2026-08-01 and returned no qualifying source (see §2.2); NHANES III microdata acquired and its variables confirmed present (§2.3).

1. Prior-observation disclosure (read this first)

Pre-registration is worthless if it is written after the result is known and does not say so.

The following was **already observed** during feasibility scoping on 2026-07-26, before this document existed:

> ***In NHSBSA FOI-02868, England 2023/24 child orthodontic starts by IOTN band were***
>
> ***22 / 141 / 8,273 / 154,170 / 35,665 for bands 1–5, i.e. 163 of 198,271 (0.082%) below the***

NHS eligibility threshold.

Consequence, stated plainly. Estimand **E1** (the below-threshold share) is **descriptive-confirmatory, not a pre-registered test.** It is reported as an observation whose magnitude was known in advance. The manuscript must say so in Methods, not only here. Any sentence implying E1 was predicted would be false.

The analyses that are **genuinely pre-specified and unseen** are **E3** (residual unmet need) and **E4** (its social gradient). The NHANES III individual-level files have not been opened. That is where this document does real work.

Also already observed, and likewise confirmatory: the Norwegian recipient shares in SSB Rapporter 2023/37 Table 7.2 (a 0.3, b 10.5, c 6.2, examined-only 2.0, total 19.0), and the hkk KIG distribution containing no code below grade 3.

2. Data sources (frozen)

Unit	Source	Access	Role
England, Wales	NHSBSA FOI-02868, 7 CSVs + CKAN metadata, OGL-UK-3.0	Open, snapshotted	E1, E2
Norway	SSB Rapporter 2023/37 (KUHR), 2012–2022	Open, snapshotted	E1, E2
Germany	IGES Gutachten KfO (BMG-commissioned)	Open, snapshotted	E2 documentary
USA	NHANES III (1988–94) occlusal battery + treatment-receipt items	Open, public domain	E3, E4

2.0 Open-data-only constraint (decision, 2026-07-27)

This study uses only fully open, immediately public data. Sources requiring registration, application, or a licence agreement are excluded by decision, not by availability.

The material consequence: **CDHS 2013 (UK Data Service SN 7774) is excluded.** It was the only non-US source carrying pre-treatment normative need and treatment receipt on the same individuals. Estimands E3 and E4 therefore rest on **NHANES III alone**, i.e., one country, fielded 1988–94. This is a substantial narrowing, and the manuscript must state it in Methods and Limitations, not only here. It is a deliberate scope decision, recorded before any outcome was computed.

If a **fully open** source carrying pre-treatment need and treatment receipt on the same individuals is identified before analysis begins, it may be added by a documented amendment to this preregistration, timestamped before any outcome is computed. No source will be added after

an estimand has been calculated.

2.2 Amendment window exercised, 2026-08-01 - no qualifying source
The window above was exercised before any outcome was computed. **No qualifying source exists.**

- **Brazil, SB Brasil** - disqualified, request-gated. Verified live at `gov.br/saude/.../sb-brasil/dados`: the 2010 wave requires a signed form emailed to cosab@saude.gov.br; the 2023 wave requires a Google Form. Neither offers direct download.
- **Mexico, ENSANUT / SIVEPAB** - no clinical malocclusion or occlusal-trait battery in a population survey with individual-level treatment receipt.
- All other national surveys were already excluded: Colombia ENSAB IV, Korea KNHANES, Australia NCOHS/NSAOH, Germany DMS and UK CDHS require registration or application; Japan, China, India, Spain, Poland, Thailand, Malaysia and South Africa publish aggregate tables only; NHANES cycles after NHANES III dropped the occlusal exam.

This is recorded as a result, not only as a limitation. NHANES III appears to be the only fully open population source carrying a clinical occlusal battery and orthodontic treatment receipt on the same individuals. That is the same structural argument as E2 seen from the other side: the conditions for measuring over-treatment are met nowhere, and the conditions for measuring unmet need openly are met by one survey, in one country, fielded 1988-94. Both belong in the identification statement.

2.3 NHANES III variables confirmed present, 2026-08-01
Confirmed from the snapshotted SAS layouts before any record was read. Occlusal battery in `exam.sas`: `DEPOVJET` (overjet, mm), `DEPOVB` (overbite, mm), `DEPOPB` (openbite), `DEPXBITE` (posterior crossbite), `DEPDIAS` (maxillary diastema), `DEPUAL1`-`DEPUAL5` (anterior alignment, mm). Treatment receipt: `HYF7` (youth) and `HAQ8` (adult), "Ever received orthodontic treatment". Deprivation indicator for E4: `DMPPIR`, poverty income ratio.

Extraction rule for FOI-02868 Part 1 (schema verified 2026-07-27): a three-row header — row 0 measure group (`UNIQUE_PATIENT_STARTS_WITH_ASSESS_&_APPLIANCE_FITTED` vs `UNIQUE_PATIENT_COMPLETIONS`), row 1 age group (`ADULT`/`CHILD`), row 2 IOTN band

(`IOTN_1`…`IOTN_5`, `IOTN_NOT_APPLICABLE`, `N/A`), over 12 data rows: 6 financial years (2018/2019–2023/2024) × 2 nations (England, Wales). Columns are selected by the (measure, age, band) triple, never by positional index.

2.1 The completions prohibition

Only the STARTS columns may be treated as needed. The COMPLETIONS columns are post-treatment residual occlusal status: child starts are 0.1% at IOTN 1–2 while child completions are 86.6%, and the NHSBSA field schema requires an IOTN score *at the completion of treatment*. Using completions as need would invert the estimand. This is a **stop condition**, not a caution: any analysis script that reads a COMPLETIONS column into a need variable is a defect to be fixed, not interpreted.

3. Estimands

E1 - Below-threshold treated share *(descriptive-confirmatory; see §1)*

Per country-unit and year: the proportion of publicly funded orthodontic treatment starts recorded below the system's own eligibility threshold. England/Wales: IOTN DHC 1–2 as a share of bands 1–5. Norway: reported as the band composition of reimbursement recipients; no untreated denominator exists, so no need; prevalence may be derived from it.

> ***Reporting constraint. E1 is not an over-treatment estimate and must never be labelled one.***
>
> ***Its expectation under enforcement is approximately zero. Reporting it without §4's identification***
>
> ***statement attached would be actively misleading.***

E2 - Formal identification statement *(the paper's central contribution)*

An enumeration of the conditions under which orthodontic over-treatment *is* identified from a data source, and a demonstration that no currently available source satisfies them. Pre-specified conditions:

1. Need is measured **before** treatment, on the same individuals;
2. The sampling frame includes individuals who received treatment **and** those who did not;
3. Need measurement is **not conditioned on** treatment eligibility or reimbursement;
4. Treatment receipt is observed **irrespective of payer**, including private and DTC.

Pre-specified conclusion to be tested against each source: administrative sources fail (3);

population surveys measuring occlusion at examination fail (1); need-gated registers additionally fail (4). **If any source satisfies all four, E2 is falsified, and the paper's central claim fails.** That is the falsification criterion, and it is reportable either way.

E3 - Residual unmet need *(genuinely pre-specified, unseen; NHANES III only)*

The proportion of children with normative need present who have **neither** received orthodontic treatment **nor** are wearing an appliance at examination. Reported using NHANES III occlusal characteristics **only**, the manual states there is no summary TPI number, and none will be constructed. Because a single instrument in a single country now carries this estimand, no cross-national comparison of E3 is possible, and none will be attempted.

E4 - Social gradient in unmet need *(genuinely pre-specified, unseen; NHANES III only)*

The association between E3 and the NHANES III poverty-income ratio, reported as a risk difference and a relative index of inequality with 95% intervals.

4. Model specification

One hierarchical logistic model, fitted to NHANES III only. No pooling across units arises, because only one unit now contributes E3/E4. The instruments across units (IOTN; Norwegian HELFO bands a/b/c; NHANES III occlusal characteristics) remain non-commensurable, and no crosswalk between them is claimed or used anywhere in this study.

`

unmet_i ~ Bernoulli(p_i)

logit(p_i) = alpha + beta_pir *poverty_income_ratio_i + beta_age* age_i + beta_sex sex_i + u_psu[i]

u_psu ~ Normal(0, sigma_psu^2)

`

Survey weights and the complex design (stratum, PSU) are respected; weighted estimation with design-based variance, or a design-informed Bayesian model with the PSU random effect above.

Priors (declared before fitting, per `docs/PRIORS.md`): `alpha ~ Normal(0, 1.5)`; `beta_ ~ Normal(0, 1)` on the logit scale, weakly informative, admitting odds ratios to roughly 7-fold; `sigma_psu ~ HalfNormal(1)`.

Convergence, non-negotiable: R-hat < 1.01, ESS_bulk > 400 per parameter, zero post-adaptation divergent transitions. Failing any of these is a blocker, not a footnote. Posterior predictive checks committed to `08_outputs/ppc/`.

Missing data: missing stays missing. Covariate missingness is handled within the model; no imputation of the outcome. The missingness rate is reported per variable.

Seed: fixed and recorded in provenance.

5. Pre-committed stop conditions

Numeric thresholds are fixed **now**, before the corresponding result is seen. Under P6 they may not be revised afterwards. Each requires a human countersignature at G1.

#	Condition	Consequence
S1	Any analysis script reads a FOI-02868 COMPLETIONS column into a need variable	Defect. Fix and re-run; the result is void
S2	Any source satisfies all four E2 identification conditions	**E2 is falsified.** Report it; the central claim fails
S3	R-hat ≥ 1.01, or ESS_bulk < 400, or ≥ 1 post-adaptation divergence	Blocker. Do **not** add iterations and re-run hoping (P10)
S4	**FIRED BY DECISION 2026-07-27**, open-data-only constraint excludes CDHS 2013	E3/E4 are reported for the US alone. Recorded here rather than removed, so the condition and its trigger remain auditable
S5	Appliance-status or prior-treatment coding is absent or ambiguous in a unit	That unit contributes to E1/E2 only, never to E3/E4
S6	Unweighted and design-weighted E3 estimates differ by > 5 percentage points	Report both; investigate; do not silently prefer either
S7	Any headline number lacks a `.prov.json` resolving to a MANIFEST entry	No submission

6. Contamination diagnostics (registered before fitting)

The most likely undetected error is **treated-status contamination**: including individuals whose occlusion has already been altered by treatment, which biases measured need downward by approximately the treatment prevalence itself (~9% at age 12 and ~18% at age 15 in England, Wales and Northern Ireland in 2013, per Rolland et al.,[20] *Br Dent J* 2016;221(7):415–419).

Two fields are extracted for **every** record, and one refit is registered:

5. `appliance_status` ∈ {absent, present, not stated}
6. `prior_treatment` ∈ {excluded, included, not stated}

Registered diagnostic: a leave-one-out refit restricted to records coded `prior_treatment = excluded`. If the deprivation coefficient moves by more than 0.2 on the logit scale **between the full and restricted fits, the gradient is reported as** contamination-sensitive and the restricted fit becomes primary. This threshold is fixed now.

7. What this study does not claim

Carried from `docs/PRIOR_ART.md`, which is binding:

- **Not** the first to distinguish normative from felt or expressed need (Sheiham 1982).[16]
- **Not** the first to quantify orthodontic demand exceeding normative need (Turbill 1996, 1998; HHS OIG 2015),[2-4] nor to frame two-sided misallocation (Watt 2019).[1]
- **Not** the first to analyse need-based allocation under need-criterion reimbursement (Jiang et al. 2024),[5] that national-scale null is engaged directly, not assumed away.
- **No** global or worldwide claim. Five country-units, three sovereign states. The words "global" and "living" must not appear in the title or abstract.
- **No** composite index. Components are reported separately; no scalar is formed, and Methods states why.

8. Deviations

Any departure from this document is recorded in `state/FINDINGS.md` with the pre-registered expectation alongside the observed result, and in the manuscript's deviations table. Widening a search, relaxing a threshold, or dropping a unit to obtain a result is a **P10 violation** and requires a logged blocker and a stop, not a silent adjustment.

9. Signature block

To be completed by a human at gate G1 (`state/GATES/G1.yaml`). The agent may not self-certify.

- [ ] Zenodo deposition published; DOI recorded
- [ ] Author list, affiliations and ORCIDs confirmed
- [ ] Stop conditions S1–S7 reviewed and accepted
- [ ] §6 diagnostic threshold (0.2 logit) reviewed and accepted
- [ ] §1 prior-observation disclosure reviewed and accepted as complete and accurate

10. Amendment A-01 - S5 disposition and the E3 need definition

Registered 2026-08-02. Decided by the project lead before any E3 or E4 result was computed.

No estimate of unmet need, and no gradient, had been produced at the time of this decision; the extraction (T-04-002) had produced sample sizes and treatment prevalence only. Amending now is therefore permitted; amending after seeing E3 would be a P6 violation.

A-01.1 Stop condition S5, resolved by the superset reading

S5 fires on its appliance-status limb: NHANES III carries no appliance-at-examination field in

any of the exam, youth, or adult layouts (zero matches for `APPL|ORTH|BRACE|BAND|RETAIN`), and the dental manual directs that teeth "banded or bracketed for orthodontic treatment are examined in the usual manner", so a person in active treatment is measured mid-treatment with no flag.

Decision: the appliance clause of E3 is redundant, not missing. `HYF7` asks whether the person ever received orthodontic treatment "such as wearing braces, bands, or removable appliances to straighten teeth". Anyone wearing an appliance at examination has necessarily answered yes. Therefore

{wearing an appliance at examination} ⊆ {ever received orthodontic treatment}

and consequently `{HYF7 = No}` = {never treated **and** no appliance}. E3 is computable as the proportion with need present among those coded `prior_treatment = excluded`. E3 and E4 proceed.

Residual risk, stated: `HYF7` (and `HAQ8` for ages 17+) is self- or proxy-reported. A person who under-reports past treatment is misclassified as untreated, biasing measured need downward among the "untreated". This is a measurement limitation, not an identification failure, and it is reported as such. It does not affect E2.

A-01.2 The E3 need definition - an any-of composite of published cut-points

`docs/THRESHOLDS.md` as signed at G1 mapped NHS IOTN DHC 4–5 to "E1, E3". That mapping was defective: NHANES III records no IOTN, and §4 of this document forbids any crosswalk between instruments. It is corrected here rather than silently used or silently dropped.

Cut-points are taken from the NIDR/NCHS analysis of **these same NHANES III variables** — Brunelle JA, Bhat M, Lipton JA, *J Dent Res* 1996;75 Spec No:706–13, PMID 8594094,[7] DOI 10.1177/002203459607502S10,[7] snapshotted at `00_snapshot/literature/pubmed_8594094_brunelle_1996.xml`. They are therefore the instrument's own published severity categories, not a foreign index mapped onto it.

`need_present = 1` if **any** of the following holds:

#	Criterion	Variable	Source
N1	overjet ≥ 6 mm	DEPOVJET	Brunelle 1996: "less than 10% had overjet of 6 mm or more"
N2	overbite ≥ 6 mm	DEPOVB	Brunelle 1996: "severe overbite of 6 mm or more"
N3	open bite present	DEPOPB measured	Manual §5.9.2: overbite and open bite are mutually exclusive conditions; a measurement is recorded only when open bite is present
N4	posterior crossbite present	`DEPXBITE = 1`	Native binary coding

N5	maxillary diastema ≥ 2 mm	`DEPDIAS = 1`	Native coding; Brunelle 1996 reports at this cut

Incisor irregularity is deliberately EXCLUDED from the primary composite. Little RM, *Am J Orthod* 1975;68(5):554–63, PMID 1059332, DOI 10.1016/0002-9416(75)90086-x,[10] cited in the NHANES III dental manual's own reference list and snapshotted at `00_snapshot/literature/pubmed_1059332_little_1975.xml`, defines the index but publishes no severity bands. The Brunelle abstract does report a 6 mm displacement cut ("11% and 15% of persons had 6 mm or more displacement of maxillary and mandibular *molars*"), but that sentence says *molars* while sitting in a passage otherwise concerned with incisors, and NHANES III's occlusal battery measures anterior segments only (`DEPUAL1–5`, `DEPLAL1–5`). The published wording is ambiguous and is not resolved by assumption.

- **Primary composite:** N1–N5 above.
- **Pre-specified sensitivity composite (SC-1):** N1–N5 **or** summed anterior irregularity ≥ 6 mm, computed separately for the maxillary and mandibular segment sums. Reported alongside the primary, never in place of it.
- Every component is additionally reported on its own, per the manual's own advice that "the disaggregated information of components of occlusion like overjet and overbite are quite useful".

Direction of bias, declared in advance. The primary composite omits incisor irregularity (crowding) and does not capture reverse overjet, since the published cut is one-sided (≥ 6 mm). Both omissions remove people who would generally be regarded as having need. E3 is therefore a lower bound on need, and the resulting unmet-need estimate is conservative by construction. Any reader who prefers a wider definition should read SC-1 and the component table.

A-01.3 Reconciliation check on the extraction

Brunelle (1996)[7] reports, weighted, that "almost 20% of adults ages 18–50, as well as 18% of children, have had orthodontic treatment". The T-04-002 extraction reproduces 18.5% and 17.7% respectively using `WTPFEX6`. This is recorded as an independent external check that the fixed-width parse, the youth/adult join, and the weighting are correct.
