# Supplementary material for: When Can Orthodontic Over-Treatment Be Measured? A Pre-Registered Identification Assessment, a Falsified Claim, and a Cross-Sectional Analysis of Untreated Occlusal Need in NHANES III
Source: Healthcare (Basel). 2026 Oct 12;14(20):3395. doi: 10.3390/healthcare14203395 (PMC13704488; doi:10.3390/healthcare14203395)
Supplement: Supplementary file 1 [file healthcare-14-03395-s001.zip › File_S3_Amendment_A01.pdf]

Supplementary File S3. Registered amendment A-01

Decided before any unmet-need result had been computed.

10. Amendment A-01 - S5 disposition and the E3 need definition

Registered 2026-08-02. Decided by the project lead before any E3 or E4 result was computed.

No estimate of unmet need, and no gradient, had been produced at the time of this decision; the extraction (T-04-002) had produced sample sizes and treatment prevalence only. Amending now is therefore permitted; amending after seeing E3 would be a P6 violation.

A-01.1 Stop condition S5 - resolved by the superset reading

S5 fires on its appliance-status limb: NHANES III carries no appliance-at-examination field in any of the exam, youth or adult layouts (zero matches for `APPL|ORTH|BRACE|BAND|RETAIN`), and the dental manual directs that teeth "banded or bracketed for orthodontic treatment are examined in the usual manner", so a person in active treatment is measured mid-treatment with no flag.

Decision: the appliance clause of E3 is redundant, not missing. `HYF7` asks whether the person ever received orthodontic treatment "such as wearing braces, bands, or removable appliances to straighten teeth". Anyone wearing an appliance at examination has necessarily answered yes. Therefore

$$\{\textit{wearing an appliance at examination}\} \subseteq \{\textit{ever received orthodontic treatment}\}$$

and consequently {`HYF7 = No`} = {never treated **and** no appliance}. E3 is computable as the proportion with need present among those coded `prior_treatment = excluded`. E3 and E4 proceed.

Residual risk, stated: `HYF7` (and `HAQ8` for ages 17+) is self- or proxy-reported. A person who under-reports past treatment is misclassified as untreated, biasing measured need downward among the "untreated". This is a measurement limitation, not an identification failure, and it is reported as such. It does not affect E2.

A-01.2 The E3 need definition - an any-of composite of published cut-points

`docs/THRESHOLDS.md` as signed at G1 mapped NHS IOTN DHC 4–5 to "E1, E3". That mapping was defective: NHANES III records no IOTN, and §4 of this document forbids any crosswalk between instruments. It is corrected here rather than silently used or silently dropped.

Cut-points are taken from the NIDR/NCHS analysis of **these same NHANES III variables** — Brunelle JA, Bhat M, Lipton JA, *J Dent Res* 1996;75 Spec No:706–13, PMID 8594094, DOI 10.1177/002203459607502S10,[7] snapshotted at `00_snapshot/literature/pubmed_8594094_brunelle_1996.xml`. They are therefore the instrument's own published severity categories, not a foreign index mapped onto it.

`need_present = 1` if **any** of the following holds:

#	Criterion	Variable	Source
N1	overjet ≥ 6 mm	DEPOVJET	Brunelle 1996: "less than 10% had overjet of 6 mm or more"
N2	overbite ≥ 6 mm	DEPOVB	Brunelle 1996: "severe overbite of 6 mm or more"
N3	open bite present	DEPOPB measured	Manual §5.9.2: overbite and open bite are mutually exclusive conditions; a measurement is recorded only when open bite is present
N4	posterior crossbite present	`DEPXBITE = 1`	Native binary coding
N5	maxillary diastema ≥ 2 mm	`DEPDIAS = 1`	Native coding; Brunelle 1996 reports at this cut

Incisor irregularity is deliberately EXCLUDED from the primary composite. Little RM, *Am J Orthod* 1975;68(5):554–63, PMID 1059332, DOI 10.1016/0002-9416(75)90086-x[10] — cited in the NHANES III dental manual's own reference list and snapshotted at `00_snapshot/literature/pubmed_1059332_little_1975.xml` — defines the index but publishes no severity bands. The Brunelle abstract does report a 6 mm displacement cut ("11% and 15% of persons had 6 mm or more displacement of maxillary and mandibular *molars*"), but that sentence says *molars* while sitting in a passage otherwise concerned with incisors, and NHANES III's occlusal battery measures anterior segments only (`DEPUAL1–5`, `DEPLAL1–5`). The published wording is ambiguous and is not resolved by assumption.

- **Primary composite:** N1–N5 above.
- **Pre-specified sensitivity composite (SC-1):** N1–N5 **or** summed anterior irregularity ≥ 6 mm, computed separately for the maxillary and mandibular segment sums. Reported alongside the primary, never in place of it.
- Every component is additionally reported on its own, per the manual's own advice that "the disaggregated information of components of occlusion like overjet and overbite are quite useful".

Direction of bias, declared in advance. The primary composite omits incisor irregularity (crowding) and does not capture reverse overjet, since the published cut is one-sided (≥ 6 mm).

Both omissions remove people who would generally be regarded as having need. E3 is therefore a lower bound on need, and the resulting unmet-need estimate is conservative by construction. Any reader who prefers a wider definition should read SC-1 and the component table.

A-01.3 Reconciliation check on the extraction

Brunelle (1996)[7] reports, weighted, that "almost 20% of adults ages 18–50, as well as 18% of children, have had orthodontic treatment". The T-04-002 extraction reproduces 18.5% and 17.7% respectively using `WTPFEX6`. This is recorded as an independent external check that the fixed-width parse, the youth/adult join, and the weighting are correct.
