# Supplementary material for: When Can Orthodontic Over-Treatment Be Measured? A Pre-Registered Identification Assessment, a Falsified Claim, and a Cross-Sectional Analysis of Untreated Occlusal Need in NHANES III
Source: Healthcare (Basel). 2026 Oct 12;14(20):3395. doi: 10.3390/healthcare14203395 (PMC13704488; doi:10.3390/healthcare14203395)
Supplement: Supplementary file 1 [file healthcare-14-03395-s001.zip › File_S4_STROBE_mapping.pdf]

Supplementary File S4. STROBE compliance mapping

STROBE compliance mapping - cross-sectional study

How to use this document. The item numbers below follow the STROBE checklist for cross-sectional studies. **The short labels are ours, not the official STROBE wording**, which is copyrighted and is not reproduced here. Download the official checklist from <https://www.strobe-statement.org/checklists/> and paste the compliance column below into it; the numbering is aligned so the transfer is mechanical.

Section references are to `08_outputs/manuscript.rendered.md`.

#	Item (our short label)	Addressed	Where
1a	Design indicated in title or abstract	Yes	Abstract, Materials and Methods paragraph: cross-sectional analysis of a national examination survey, plus a documentary assessment of four data sources
1b	Informative, balanced abstract	Yes	Structured abstract: Background and Objectives / Materials and Methods / Results / Conclusions
2	Background and rationale	Yes	§1, paragraphs 1–3
3	Objectives and pre-specified hypotheses	Yes	§1, paragraphs 4–5; the four identification conditions and the falsification rule are stated in §2.2
4	Study design early in the paper	Yes	§2.1–2.2
5	Setting, locations, relevant dates	Yes	§2.3 (four country-units); §2.4 (NHANES III, 1988–1994, ages 8–17)
6a	Eligibility criteria, sources, selection methods	Yes	§2.3 for source selection; §2.4 for the analysed sample (children 8–17 who received the occlusal examination)
7	Variables: outcomes, exposures, predictors, confounders	Yes	§2.5 (need composite and its two sensitivity forms); §2.6 (treatment receipt); §2.7 (poverty income ratio, age, sex)
8	Data sources and measurement methods per variable	Yes	§2.4 (sentinel handling, layout parsing); §2.5 (cut-point provenance); §2.6 (treatment-receipt item)
9	Efforts to address sources of bias	Yes	§2.6 (appliance status and the treatment-receipt superset); §2.7 (registered contamination diagnostic); §4.3
10	Study size	Yes	§3.3 and §3.4 report the examined, need-present and analysed counts. No a priori size calculation: the sample is the entire eligible survey stratum, so size was not chosen
11	Handling of quantitative variables and groupings	Yes	§2.5 (cut-points and composites); §2.7 (poverty income ratio used continuously in the primary model and as weighted fractional rank for the inequality indices); §3.5 (quintiles descriptive only)
12a	Statistical methods including confounding control	Yes	§2.7
12b	Methods for subgroups and interactions	Yes	§2.7 and §3.5: the need/access decomposition is the only stratified analysis; no interaction terms were fitted
12c	Handling of missing data	Yes	§2.7 (missing left missing, no outcome imputation); §3.4 reports the missingness rate and compares the outcome between excluded and analysed records
12d	Sampling strategy accounted for	Yes	§2.7: examination weight as a normalised pseudo-likelihood weight, and a pseudo-stratum by pseudo-primary-sampling-unit random effect
12e	Sensitivity analyses	Yes	§2.5 (composites SC-1 and SC-2); §2.7 and §3.4 (registered contamination refit); §3.3 reports the composite range
13a	Numbers at each stage	Yes	§3.3 (examined), §3.4 (need present, analysed, excluded for missing covariate)
13b	Reasons for non-participation	Partial	The number excluded for a missing poverty income ratio is reported with the survey's own reason code. Reasons for survey non-response are a property of NHANES III and are documented by the survey, not by this study
13c	Flow diagram	No	Not included. Sample derivation is a two-step restriction stated in text (age band, then occlusal examination received); a diagram was judged to add nothing. Available on request
14a	Participant characteristics	Partial	Age band, sex and the poverty income ratio distribution are used throughout; a conventional Table 1 of participant characteristics is not presented because the paper's tables are given to the identification matrix and the estimands
14b	Missing data per variable	Yes	§3.4 for the poverty income ratio; Table 3 and the component table report per-variable "could not be assessed" counts

15	Outcome events or summary measures	Yes	§3.3–3.4; Table 2; Table 3
16a	Unadjusted and adjusted estimates with precision	Yes	§3.4 and §3.5 report weighted descriptive prevalences alongside the model-based standardised risks with 95% credible intervals. Table 2
16b	Category boundaries when continuous variables categorised	Yes	§2.5 (need cut-points); §3.5 (quintile boundaries defined on weighted fractional rank)
16c	Absolute risk where relevant	Yes	§3.5 reports the risk difference in percentage points alongside the relative index of inequality, and §3.5 states explicitly that the two scales order the decomposition limbs differently
17	Other analyses	Yes	§3.5 (three-limb decomposition on three scales); §3.4 (contamination refit)
18	Key results with reference to objectives	Yes	§4.1; §5
19	Limitations	Yes	§4.3, including single country-unit, survey vintage, definitional sensitivity, self-report, and the scale-dependence of the decomposition
20	Cautious overall interpretation	Yes	§4.1 and §4.4
21	Generalisability	Yes	§4.3: the estimate is explicitly stated to be non-contemporary and non-transportable, and no cross-national comparison is made
22	Funding	Yes	Funding section: no external funding

Items where this study departs from a conventional cross-sectional report

Two departures are worth an editor's attention rather than being buried in the table.

No flow diagram (13c) and no conventional Table 1 (14a). The paper's principal result is an identification argument rather than a participant-level comparison, and the three table slots were given to the identification matrix, the estimands and the need components. Both items can be supplied if the editor prefers.

Study size (10) was not chosen. The analysed sample is the complete eligible stratum of a national survey. There was no recruitment and no power calculation, and the precision reported is whatever that stratum supports.
