# Supplementary material for: When Can Orthodontic Over-Treatment Be Measured? A Pre-Registered Identification Assessment, a Falsified Claim, and a Cross-Sectional Analysis of Untreated Occlusal Need in NHANES III
Source: Healthcare (Basel). 2026 Oct 12;14(20):3395. doi: 10.3390/healthcare14203395 (PMC13704488; doi:10.3390/healthcare14203395)
Supplement: Supplementary file 1 [file healthcare-14-03395-s001.zip › File_S5_GATHER_mapping.pdf]

Supplementary File S5. GATHER compliance mapping

GATHER compliance mapping - health-estimate reporting

How to use this document. GATHER (Guidelines for Accurate and Transparent Health Estimates Reporting) comprises 18 items across five domains. The domain and item labels below are ours, not the official GATHER wording, which is not reproduced here. Download the official checklist from <http://gather-statement.org/> or the EQUATOR Network and paste the compliance column into it.

An honest caveat on this document. The official item wording could not be retrieved programmatically while this package was assembled, so the item-number alignment below should be checked against the downloaded template before submission rather than trusted blindly. The compliance evidence itself is accurate and each entry points at a real artifact; it is only the mapping of evidence to item *numbers* that warrants a check.

Section references are to `08_outputs/manuscript.rendered.md`. Repository paths are relative to the archived study repository.

Domain 1 - Objectives and funding (items 1–2)

#	Item (our short label)	Compliance
1	Define the indicator, population and time period	Estimands are defined in Section 2.2 (the identification conditions), Section 2.5 (need) and Section 2.6 (unmet need). Population: United States children aged 8–17 examined in NHANES III, 1988–1994. The identification assessment covers four country-units. All were registered before analysis in `docs/PREREG_C.md` Section 3
2	List funding sources	Funding section: no external funding. No funder had any role

Domain 2 - Data inputs (items 3–6)

#	Item (our short label)	Compliance
3	How data were identified and obtained	`config/sources.yaml` records every source with its retrieval URL. `scripts/fetch.py` performs retrieval and records HTTP status, UTC timestamp, byte size and SHA-256 in `00_snapshot/MANIFEST.jsonl`. A non-200 response writes a failure record and never a substituted payload
4	Inclusion and exclusion criteria	Section 2.1 (open-data-only restriction, applied as a registered stop condition); Section 2.3 (source selection); Section 2.4 (age band and receipt of the occlusal examination); Section 2.7 (complete cases on the poverty income ratio)
5	Information on all data sources	Section 2.3 and Section 2.4; full detail with checksums in `00_snapshot/MANIFEST.jsonl` and in the G6 audit packet `08_outputs/g6_claim_audit.md`
6	Sources not publicly available, and why	None were used. The open-data-only restriction excluded every registration-, application- or licence-gated source, including one national child dental health survey. That exclusion is recorded as stop condition S4 in the registration and is stated in Section 2.1 and Section 4.3. This is a *consequence* reported as a result, not an omission

Domain 3 - Data processing (item 7)

#	Item (our short label)	Compliance

7	Steps taken to process data	Section 2.4: sentinel codes for "could not be assessed" mapped to typed missing-reason fields and never read as measurements; the poverty-income-ratio blank-but-applicable code likewise excluded; fixed-width column positions parsed from each file's own layout specification because the same variable occupies different columns in different files. Every extraction step is reconciled against the survey codebook's published marginal counts at run time, and the extraction refuses to emit if the reconciliation fails

Domain 4 - Analysis and modelling (items 8–10)

#	Item (our short label)	Compliance
8	Description or diagram of analysis steps	Section 2.5–2.7. The full task sequence, with dependencies, is recorded in `state/TASKS.yaml`; each analysis artifact carries a `.prov.json` naming the code that produced it and the inputs it consumed
9	Details of all models	Section 2.7: hierarchical logistic regression, model form, all priors, the survey-weight treatment, the random-effect structure, and the population-standardisation procedure for the inequality indices. Model code is `scripts/analysis/t04_003_unmet_need.stan`. Registered convergence criteria and their observed values are reported in Section 3.4
10	Sources of code	All analysis code is archived with the study repository. Every number in the manuscript resolves through `08_outputs/manuscript.claims.json` to the code file and task that produced it

Domain 5 - Results and discussion (items 11–18)

#	Item (our short label)	Compliance
11	Results with uncertainty	Section 3.4–3.5 and Table 2: posterior means with 95% credible intervals for every model-based quantity. Uncertainty is propagated as posterior draws, not summaries — the inequality indices are computed per draw and summarised only at the end
12	Comparison with previous estimates	Section 3.3: the weighted proportion of children ever treated reproduces the published estimate from the same survey, and the published figures are extracted from the archived record rather than transcribed. Section 4.2 relates the identification result to the prior literature, including a national register study reaching an apparently opposite conclusion
13	Limitations	Section 4.3, and additionally Section 3.3 (definitional sensitivity of the need composite) and Section 3.5 (scale-dependence of the decomposition)
14	Differences from any pre-specified analysis plan	Reported explicitly. One registered amendment (A-01) resolved an absent appliance-status field and supplied the need cut-points that the registration had left unspecified; it was registered before any unmet-need value was computed and is described in Section 2.5–2.6 and archived in full. Sensitivity composite SC-2 was added after the component distribution was observed but before any unmet-need value existed, and is labelled as such in Section 2.5
15	Statement on ethics approval or waiver	Institutional Review Board Statement: waived; de-identified public-use secondary data and published aggregate statistics
16	Data availability	Data Availability Statement: all primary sources are public and unrestricted; each is recorded with URL, timestamp, size and SHA-256
17	Conflicts of interest	Conflicts of Interest section: none declared
18	Reproducibility of estimates	Every estimate is reproducible from the archived code and the checksummed source files. Random seeds are fixed and recorded in provenance. The templating harness refuses to render an unresolved value, so no number in the text can exist without a provenance record

What a reader should check first

The single most consequential judgement in this study is not statistical. It is whether the four identification conditions in Section 2.2 are the right four. They are this paper's proposal rather than an established framework, they were fixed before any source was assessed, and the manuscript says so. A reader who disagrees with them should disagree with the paper's central claim, and that is the intended failure mode.
