# Supplementary material for: When Can Orthodontic Over-Treatment Be Measured? A Pre-Registered Identification Assessment, a Falsified Claim, and a Cross-Sectional Analysis of Untreated Occlusal Need in NHANES III
Source: Healthcare (Basel). 2026 Oct 12;14(20):3395. doi: 10.3390/healthcare14203395 (PMC13704488; doi:10.3390/healthcare14203395)
Supplement: Supplementary file 1 [file healthcare-14-03395-s001.zip › File_S6_Software_and_tools.pdf]

File S6 - software and tools

Versions read from the environment.

File S6 - software and tools

Every version below is read from the running environment, not typed. The revision round added the R `survey` package (design-based estimation, Section 2.9) and `mitools`; everything else is as at submission.

Component	Version
R	4.3.3
R:ggdist	3.3.3
R:ggplot2	4.0.2
R:mitools	2.7
R:patchwork	1.3.2
R:ragg	1.5.0
R:survey	4.5
R:svglite	2.2.2
R:systemfonts	1.3.1
cmdstan	2.39
cmdstanpy	1.3.0
matplotlib	3.11.1
numpy	2.5.1
pandas	3.0.5
platform	Linux-6.17.0-29-generic-x86_64-with-glibc2.39
polars	1.43.2
python	3.12.3
statsmodels	0.14.6

Analysis code, the registered protocol and both amendments are archived under concept DOI 10.5281/zenodo.21725476.
