# Supplementary material for: When Can Orthodontic Over-Treatment Be Measured? A Pre-Registered Identification Assessment, a Falsified Claim, and a Cross-Sectional Analysis of Untreated Occlusal Need in NHANES III
Source: Healthcare (Basel). 2026 Oct 12;14(20):3395. doi: 10.3390/healthcare14203395 (PMC13704488; doi:10.3390/healthcare14203395)
Supplement: Supplementary file 1 [file healthcare-14-03395-s001.zip › File_S8_Documented_search.pdf]

File S8 - the documented data-source search

Reviewer-requested, post-hoc. Declared in registered amendment A-02.

File S8 - the documented data-source search

Reviewer-requested, post-hoc, not pre-registered. Declared in advance in registered amendment A-02 Section 8, concept DOI `10.5281/zenodo.21725476`.

Reviewers 1, 2 and 3 each objected that the manuscript's central claim is a universal negative resting on four convenience-selected sources, and asked for a systematic, reproducible search. This is that search.

Its headline result is that it falsified the paper's central claim. See Section 5.

Contents

Component	File
Search protocol, pre-specified and dated before any query, with amendments 1–4	`docs/T1_SEARCH_PROTOCOL.md`
Screening decision rule, pre-specified and dated before any record was screened	`docs/T1_SCREENING_RULE.md`
Full request log — 112 requests, each with endpoint, verbatim query, UTC timestamp, HTTP status, result count **and the universe searched**	`08_outputs/tables/t1_search_log.csv`
Retrieved corpus, 15,341 records	`08_outputs/tables/t1_records.csv`
Screening log, one row per record with its decision and typed exclusion code	`08_outputs/tables/t1_screening_log.csv`
PRISMA counts, computed from the log and reconciling	`08_outputs/tables/t1_prisma_counts.json`
The falsification finding and its verification	`state/FINDINGS.md`, `docs/S2_FALSIFICATION_BRIEFING.md`

1. What was searched

Eight source frames, fixed in the protocol before execution: OECD national statistical office catalogues; health-ministry open-data portals; WHO Global Health Observatory; the Global Oral Health Data Portal; national dental survey series; open government data portals; PubMed; and a bibliographic frame that became OpenAlex and Europe PMC by amendment.

2. What was not searched, and why

Declared rather than omitted. No substitute is claimed to cover any of these.

Frame	Status
**Scopus**	Withdrawn by amendment 1 and replaced with keyless OpenAlex and Europe PMC. A search behind a subscription API is reproducible only for subscribers, the same defect the paper identifies in need-gated records.
**data.gov**	Hard 404 at every documented endpoint, and not a Socrata domain. The HHS catalogue healthdata.gov *was* searched (amendment 4).
**Germany GOVDATA**	DNS resolves; TCP to :443 never completes from this environment. Unreachable here, not absent.

3. Counts

**Requests logged**	**112**

Records identified	15,341
Duplicates removed	1,202
Screened at title/abstract	14,139
Excluded	12,908
Referred to full text	1,065
Included	166, naming 117 distinct candidate sources

Counts are computed from the screening log and reconcile; they are not asserted.

4. Screener reliability

Screening was single-screener, which is a limitation. It is reported with a measured error rate rather than an assumed one: a random 10% (1,412 records, seed 20260802) was re-screened blind, giving 2.44% disagreement (33 of 1,355 comparable).

5. The result: stop condition S2 fired

Of 26 candidates assessed against I1–I4 using each custodian's own documentation, 25 fail, and they fail exactly where the paper predicts, population surveys on I1, administrative sources on I3. Two *open* sources sit at 3 of 4, the Canadian Health Measures Survey and NHANES III itself, both failing only I1.

One satisfies all four. The US National Health Examination Survey, Cycles II (1963–65, ages 6–11) and III (1966–70, ages 12–17), NCHS. It does so because it is two linked cycles: NCHS distributes a person-level Cycle II linkage key in the open public-use release, giving a pre-adolescent occlusal record for part of the Cycle III sample.

Verified against the tapes, not only the documentation: the key is populated exactly as the codebook states (2,177 / 4,591 / 6,768); all 2,177 join; Cycle II carries both the TPI-derived malocclusion field and an orthodontic appliance field, each matching its documented distribution. Of the 2,177, 55 were already in appliance therapy at Cycle II and a further group carried no Treatment Priority Index assignment there; excluding both, a child with no pre-treatment need measurement cannot satisfy I1 either, leaves n = 2,075. Amendment A-02 Section 2b records both exclusions; earlier wording here named only the first, which did not account for the whole difference. Access tier open, both tapes retrieved anonymously, no registration.

Recorded as a limitation of the finding: the linked subsample is representative on sex and close

on race, but not on age (12–14 is 85.4% of it against 53.2% of the frame).

Under registered stop condition S2, estimand E2 is falsified and the paper's central claim does not survive as a universal negative. The mechanism it describes is supported; the universal quantifier over it is not.

No quantity has been estimated from NHES. Establishing that the source satisfies I1–I4 completes the E2 assessment; analysing it is a separate study.

6. A provenance limitation of this file, stated rather than left to be found

The two counts in Section 5 - 26 candidates assessed, 25 failing, are transcribed from the filed amendment A-02, not derived from a machine-readable register. The per-candidate I1–I4 assessment was carried out and its verdicts are recorded in prose here, in A-02 Section 2b and in the project session log, but the per-candidate table was never persisted as an artifact. Three of the 26 are named: the falsifying source, the Canadian Health Measures Survey and NHANES III. The other 23 verdicts are not individually reproducible from this repository.

Everything else in this file is reproducible: the request log (File S8a), the retrieved corpus, the screening log (File S8b), the PRISMA counts (File S8c), and the measured screener reliability are all artifacts with checksums, and the falsification itself was verified against the NHES data tapes rather than against documentation. The limitation is confined to the two summary counts.

They are carried into the manuscript labelled `amendment_A02` rather than as derived values, and `scripts/analysis/t15_revision_values.py` states the limitation at the point it reads them. The finding was on the record in gate G7 at the time it was signed.
