# Supplementary material for: When Can Orthodontic Over-Treatment Be Measured? A Pre-Registered Identification Assessment, a Falsified Claim, and a Cross-Sectional Analysis of Untreated Occlusal Need in NHANES III
Source: Healthcare (Basel). 2026 Oct 12;14(20):3395. doi: 10.3390/healthcare14203395 (PMC13704488; doi:10.3390/healthcare14203395)
Supplement: Supplementary file 1 [file healthcare-14-03395-s001.zip › File_S9_Registration_status_register.pdf]

File S9 - registration-status register for the submitted manuscript

Reviewer-requested, post-hoc. The corrections are registered in amendment A-02.

T-11 - Post-hoc audit of the submitted manuscript

Audited document: `00_snapshot/revision_1/healthcare-4543715_submitted.docx`

SHA-256: `3a366fd2167517066b2ab8efab946f6bb3d4f441b783919444ee20d32d0d1256`

Registration documents: `docs/PREREG_C.md` (File S2, whose Section 10 is amendment A-01) and `docs/THRESHOLDS.md`

Task: T-11 · **Code:** `scripts/analysis/t11_posthoc_audit.py`

What was done

Every reported quantity in the manuscript as submitted was reconciled against the registered protocol and amendment A-01, and classified as one of `registered`, `amendment_A01`, `post_hoc_declared` or `post_hoc_undeclared`. The audited region runs from the Highlights to the reference list, excluding the Section 2.8 software inventory and the back matter. Every result-bearing numeral in that region is accounted for by a classified family; the script halts rather than emitting a partial register if any is not (Supplementary Files S9a & S9b).

Classification rests on the registered documents alone. A statement in the manuscript, a code docstring, the session log, the GATHER or STROBE mapping, or a gate file is a *disclosure*, not a *registration*, and was not treated as cover.

Result

**Classification**	**Quantity families**
`registered`	16
`amendment_A01`	4
`post_hoc_declared`	1
`post_hoc_undeclared`	8
**total**	**29**

The manuscript's declaration at Section 4.3 is not complete. It states that Supplementary Figure S1 was the only analysis that was not pre-registered.

The audit finds 7 unregistered analyses. Figure S1 is the only one declared as post-hoc. Of the other 6, 3 feed a headline claim. A further 2 computational settings are also unregistered; they are listed for completeness and are not analyses.

Separately, 1 registered estimand is ambiguous between two readings that the manuscript reports side by side. It is classified as `registered`, not post-hoc, with the ambiguity recorded rather than resolved.

What a reader was misled by is larger than the classification. The manuscript asserts registered or pre-specified status for unregistered work in 9 distinct statements, bearing on 6 of the 6 unregistered analyses. Six of those statements concern SC-2 alone, including one in the Abstract, one in a figure caption, one in the Supplementary Materials list, and one in the Abbreviations table. The classification label is bookkeeping; these statements are what a reader was misled by.

The undeclared analyses

`PH-sc2` - Sensitivity composite SC-2 (removes the diastema criterion)

- **Reported in:** Abstract; Section 2.5; Section 3.3; Figure 3B; Table S1
- **Values:** 23.4%
- **Feeds a headline claim:** YES
- **Declared in the manuscript:** PARTIAL, AND CONTRADICTED. Section 2.5 states SC-2 'was added after the component distribution was observed but before any unmet-need value was computed and is labelled as such'. It is not, in fact, labelled as such anywhere. Six other statements describe it as pre-specified or registered, including the Abstract, the Figure 3 caption, the Supplementary Materials list and the Abbreviations table, and Section 4.3 excludes it by naming Figure S1 as the only analysis that was not pre-registered.
- **Searched:** docs/PREREG_C.md: registers the primary composite (line 329) and SC-1 (line 330) only; grep 'SC-?2' returns zero hits
- **Searched:** docs/THRESHOLDS.md: registers SC-1 only (line 35); zero hits for SC-2
- **Why it matters:** The Abstract reports need as a range, '23.4% to 44.5%', and the paper states it reports the range before the point estimate. The lower bound of the headline range is an unregistered quantity. Reviewer 3 recommends promoting SC-2 to the primary estimate, so its status will be examined directly on resubmission.
- **Asserted as registered in 7 place(s):**
- C1 (paragraph 17): “Weighted need prevalence among children aged 8–17 was 33.3% (23.4% to 44.5% across pre-specified composites), of whom 87.8% had never been treated.”
- C2 (paragraph 58): “The full component set and the three registered composites, with positive counts and unweighted and weighted percentages, are given in Table S2.”
- C3 (paragraph 103): “Under the pre-specified alternatives it ranged from 23.4% under SC-2, which drops the diastema criterion, to 44.5% under SC-1, which adds incisor irregularity (Figure 3, Table S2).”
- C4 (paragraph 105): “(B) Weighted need prevalence under the registered composite and its two pre-specified sensitivity forms (SC-2, without diastema;”
- C5 (paragraph 155): “Table S1: the need components and the three registered composites;”
- C6 (paragraph 179): “SC-1, SC-2 | Pre-specified sensitivity composites 1 and 2”
- C12 (paragraph 148 (Section 4.3)): “One analysis in this paper was not pre-registered. Supplementary Figure S1, which describes need and unmet need by single year of age and sex, was added after the registered analyses were complete. It”
- **Dissent on record:** DISSENT, AND WHY IT LOSES. One of two independent verifiers classified SC-2 as post_hoc_DECLARED rather than undeclared, on the ground that Section 2.5 and the Table S1 footnote both date it accurately, so 'undeclared' asserts a concealment the document twice refutes. Both verifiers agreed it has no registration cover. The dissent loses on the arithmetic of the document: the manuscript asserts pre-specification for SC-2 in SIX places against one accurate dating, and Section 2.5's own claim

that SC-2 'is labelled as such' is itself false — it is labelled as such nowhere. One accurate sentence surrounded by six inaccurate ones does not make the status declared to a reader who did not go looking for it. Author's decision, 2026-09-15.

`PH-need-limb` - Need limb of the decomposition, all Table 2 row-2 quantities

- **Reported in:** Section 3.5; Table 2 row 2; Figure 4; Figure 5
- **Values:** 39.2, 36.3, 42.2, 27.7, 25.1, 30.4, 11.5, 6.6, 16.4, 1.42, 1.22, 1.65, −0.56, −0.81, −0.32
- **Feeds a headline claim:** YES
- **Declared in the manuscript:** NONE. The Table 2 footnote attributes all three rows to 'the design-informed Bayesian hierarchical logistic model described in Section 2.7', and Section 2.7 opens 'Our registered model is…', so the limb rows are presented as outputs of the registered model.
- **Searched:** docs/PREREG_C.md: 'decompos' 0 matches; 'need limb' 0 matches; 'access limb' 0 matches. The single 'limb' hit at line 277 is the appliance-status limb of stop condition S5, an unrelated sense
- **Searched:** docs/THRESHOLDS.md: 0 matches for all four terms
- **Searched:** docs/PREREG_C.md:164 registers 'One hierarchical logistic model'; three were fitted
- **Why it matters:** E4 registers a risk difference and an RII for E3, unmet need. The need limb is a different outcome (need present), fitted as a separate model, so it is not the registered estimand. The project's own session log concedes the point: 'Decomposing where the gradient lives was not a planned analysis.'
- **Asserted as registered in 3 place(s):**
- C7 (paragraph 131 (Table 2 footnote)): "CrI, credible interval; pp, percentage points; RII, relative index of inequality. Estimates are population-standardised contrasts between the most disadvantaged and the most advantaged weighted fracti"
- C8 (paragraph 2 (Title)): "Need-Gated Reimbursement Makes Orthodontic Over-Treatment Unobservable: A Pre-Registered Multi-Source Identification Assessment and Cross-Sectional Analysis of Unmet Need and Its Social Gradient"
- C12 (paragraph 148 (Section 4.3)): "One analysis in this paper was not pre-registered. Supplementary Figure S7, which describes need and unmet need by single year of age and sex, was added after the registered analyses were complete. It"

`PH-access-limb` - Access limb of the decomposition, all Table 2 row-3 quantities

- **Reported in:** Section 3.5; Table 2 row 3; Figure 4; Figure 5; Section 4.4
- **Values:** 94.7, 92.6, 96.3, 78.0, 73.3, 82.5, 16.6, 10.9, 22.5, 1.21, 1.13, 1.31, −1.79, −2.40, −1.18, 1473
- **Feeds a headline claim:** YES
- **Declared in the manuscript:** NONE, and the Table 2 footnote presents it as a registered-model output.
- **Searched:** docs/PREREG_C.md: as for the need limb
- **Searched:** docs/THRESHOLDS.md: 0 matches
- **Searched:** docs/PREREG_C.md Section 6 registers one refit conditioned on TREATMENT status; the access limb conditions on NEED status and is a different quantity
- **Why it matters:** This is the load-bearing one. Section 4.4 concludes that 'the income gradient in unmet need operates substantially through treatment receipt rather than through need', and hypothesis H3 in Section 1 asserts the gradient is 'attributable in part to differential treatment receipt conditional on need rather than to the distribution of need alone'. Both rest entirely on an unregistered decomposition, and the title describes the whole study as 'Pre-Registered'.
- **Asserted as registered in 3 place(s):**

- C7 (paragraph 131 (Table 2 footnote)): “CrI, credible interval; pp, percentage points; RII, relative index of inequality. Estimates are population-standardised contrasts between the most disadvantaged and the most advantaged weighted fracti”
- C8 (paragraph 2 (Title)): “Need-Gated Reimbursement Makes Orthodontic Over-Treatment Unobservable: A Pre-Registered Multi-Source Identification Assessment and Cross-Sectional Analysis of Unmet Need and Its Social Gradient”
- C12 (paragraph 148 (Section 4.3)): “One analysis in this paper was not pre-registered. Supplementary Figure S7, which describes need and unmet need by single year of age and sex, was added after the registered analyses were complete. It”

`PH-e4-logit` - Rank-gradient coefficients on the logit scale, all three Table 2 rows

- **Reported in:** Table 2 'Rank gradient, logit' column; Section 3.5
- **Values:** −0.84, −1.09, −0.58
- **Feeds a headline claim:** no
- **Declared in the manuscript:** NONE. Section 2.7 discloses the rank refit as a method, but Section 4.3 certifies every analysis but Figure S1 as following the registered plan.
- **Searched:** docs/PREREG_C.md: 'rank', 'ridit' and 'fractional' return ZERO matches; docs/THRESHOLDS.md: likewise, zero
- **Searched:** docs/PREREG_C.md:159-160 gives an exhaustive two-item list of E4 reporting measures, a risk difference and an RII. A logit coefficient is a third measure not in that list
- **Searched:** docs/PREREG_C.md:173 registers beta_pir on RAW poverty_income_ratio, a different parametrisation whose estimated value is −0.184, not −0.84
- **Why it matters:** The exposure scale on which −0.84 is expressed, the weighted fractional rank, is named nowhere in either registration document. The repository's own code says so explicitly: t04_004_social_gradient.py records that the logit coefficient 'is not the registered reporting form'. Both independent verifiers and the under-accusation sweep reached this verdict against an initial classification of 'registered'.
- **Asserted as registered in 2 place(s):**
- C7 (paragraph 131 (Table 2 footnote)): “CrI, credible interval; pp, percentage points; RII, relative index of inequality. Estimates are population-standardised contrasts between the most disadvantaged and the most advantaged weighted fracti”
- C12 (paragraph 148 (Section 4.3)): “One analysis in this paper was not pre-registered. Supplementary Figure S7, which describes need and unmet need by single year of age and sex, was added after the registered analyses were complete. It”

`PH-quintiles` - Unmet need by poverty-income-ratio quintile (Figure 4)

- **Reported in:** Section 3.5; Figure 4
- **Feeds a headline claim:** no
- **Declared in the manuscript:** NONE.
- **Searched:** docs/PREREG_C.md: 'quintile' 0 matches
- **Searched:** docs/THRESHOLDS.md: 0 matches
- **Why it matters:** The quintile pattern carries the claim that the gradient is not linear in rank, which is the stated justification for how the registered RII should be read. It introduces no new outcome, exposure,

model or threshold, so it is the mildest item in this list — a descriptive re-cut of a registered outcome. Reviewers 1 and 3 both engage it.

- **Asserted as registered in 1 place(s):**
- C12 (paragraph 148 (Section 4.3)): “One analysis in this paper was not pre-registered. Supplementary Figure S7, which describes need and unmet need by single year of age and sex, was added after the registered analyses were complete. It”

`PH-excluded-comparison` - Unmet need among children excluded for a missing poverty income ratio

- **Reported in:** Section 3.4
- **Values:** 27.8%
- **Feeds a headline claim:** no
- **Declared in the manuscript:** NONE.
- **Searched:** docs/PREREG_C.md:188 registers that the missingness RATE is reported per variable; it does not register an outcome comparison between included and excluded children
- **Why it matters:** Reviewer 1 challenges this exact number as insufficient to establish ignorable missingness, and T-6 was commissioned to replace it.
- **Asserted as registered in 1 place(s):**
- C12 (paragraph 148 (Section 4.3)): “One analysis in this paper was not pre-registered. Supplementary Figure S7, which describes need and unmet need by single year of age and sex, was added after the registered analyses were complete. It”

Unregistered computational settings (not analyses)

- **`PH-age-centring`** - Age centring constant (sample mean, 12.12 years) (12.12). A computational convenience, not an analysis. It changes the intercept's interpretation but no reported estimand. Listed for completeness rather than as a finding.
- **`PH-sampler-config`** - Sampler configuration: four chains, 1000 warm-up, 1000 sampling iterations (1000). A computational setting fixed by project convention rather than by the registration. Not an analysis. Listed for completeness.

Registered, but ambiguous between two reported readings

`E3-estimand` - E3 residual unmet need, BOTH reported readings (joint over all examined children, and conditional among those with need)

- **Values:** 29.2%, 87.8%
- **Registering line:** `docs/PREREG_C.md:152`
- The registered text, 'The proportion of children with normative need present who have neither received orthodontic treatment nor are wearing an appliance at examination', does not say whether the denominator is all examined children (joint, 29.2%) or only those with need (conditional, 87.8%). The ordinary reading of 'the proportion of X who Y' makes X the denominator, which is the CONDITIONAL. The project adopted the JOINT reading as primary, arguing that PREREG Section 6's registered contamination refit would be degenerate under the conditional reading. That argument is sound and contemporaneous, but it lives in a code docstring (scripts/analysis/t04_003_unmet_need.py:8-21) and in state/SESSION_LOG.md, NOT in any registered document. So, the estimand is registered; the choice between its two readings is not. Both values are reported, and neither is concealed. 87.8% is the paper's most-quoted number, appearing in the Highlights and the Abstract.

Statements in the manuscript that the register contradicts

12 found by scanning every sentence that asserts pre-registration.

- **C1** (paragraph 17, *SC-2*): “Weighted need prevalence among children aged 8–17 was 33.3% (23.4% to 44.5% across pre-specified composites), of whom 87.8% had never been treated.”
- **C2** (paragraph 58, *SC-2*): “The full component set and the three registered composites, with positive counts and unweighted and weighted percentages, are given in Table S2.”
- **C3** (paragraph 103, *SC-2*): “Under the pre-specified alternatives it ranged from 23.4% under SC-2, which drops the diastema criterion, to 44.5% under SC-1, which adds incisor irregularity (Figure 3, Table S2).”
- **C4** (paragraph 105, *SC-2*): “(B) Weighted need prevalence under the registered composite and its two pre-specified sensitivity forms (SC-2, without diastema;.”
- **C5** (paragraph 155, *SC-2*): “Table S2: the need components and the three registered composites;”
- **C6** (paragraph 179, *SC-2*): “SC-1, SC-2 | Pre-specified sensitivity composites 1 and 2”
- **C7** (paragraph 131 (Table 2 footnote), *Table 2 footnote*): “CrI, credible interval; pp, percentage points; RII, relative index of inequality. Estimates are population-standardised contrasts between the most disadvantaged and the most advantaged weighted fractional rank of the poverty income ratio, taken over the posterior of the design-informed Bayesian hier”
- **C8** (paragraph 2 (Title), *Title*): “Need-Gated Reimbursement Makes Orthodontic Over-Treatment Unobservable: A Pre-Registered Multi-Source Identification Assessment and Cross-Sectional Analysis of Unmet Need and Its Social Gradient”
- **C9** (paragraph 78 (Table 1 caption), *Table 1 caption*): “Table 1. Assessment of four openly available country-unit data sources against the four pre-registered identification conditions for over-treatment. Conditions were fixed in the pre-registration before any source was assessed.”
- **C10** (paragraph 29 (Section 1 Introduction), *Section 1 Introduction*): “Three hypotheses were examined. The first was registered before any source was assessed, together with the rule that would falsify it.”
- **C11** (paragraph 37 (Section 2.1), *Section 2.1*): “Estimands, model form, priors, decision thresholds, and stop conditions were registered before analysis, together with a disclosure of which quantities had already been observed at the time of registration. Deposition is under DOI 10.5281/zenodo.21725477, with all analysis code archived alongside it”
- **C12** (paragraph 148 (Section 4.3), *the completeness of the post-hoc declaration*): “One analysis in this paper was not pre-registered. Supplementary Figure S1, which describes need and unmet need by single year of age and sex, was added after the registered analyses were complete. It is descriptive; no inference is drawn from it, and it is labelled as post-hoc in its own caption. E”

The single place the manuscript states the position accurately is Section 2.5, which records that SC-2 “was added after the component distribution was observed but before any unmet-need value was computed”. Six other statements, including one in the Abstract, one in a figure caption, one in the Supplementary Materials list and one in the Abbreviations table, describe SC-2 as pre-specified or registered.

How this register was checked

An audit that set out to confirm the author’s own account would be worth little, so it was built to argue against itself. Every classification was put to two independent refuters instructed to overturn it: `registered` entries by testing whether the cited line names that specific outcome and that specific measure, `post_hoc_undeclared` entries by searching the registration documents in full for cover that would reverse the

verdict. Four further sweeps examined the register as a whole, completeness, over-accusation, under-accusation and internal contradictions. Fifty-six independent checks in total.

Four verdicts changed, and all four moved toward less generosity to the original classification: the rank-gradient logit coefficients, the age centring constant and the sampler configuration each moved from `registered` to unregistered, and the split treatment of E3's two readings, which had awarded a single ambiguous sentence as cover to one reading while denying it to the other, was merged into one family.

The sweep tasked with finding **over-accusation**, by building the strongest possible case that each supposedly unregistered analysis was in fact covered, withdrew none of them. No candidate registering text succeeded.

What this audit does not say

- It does not find a fabricated number. Every quantity in the register resolves to an artifact and a checksummed source file.
- **It does not establish the order in which analyses were run against the order in which their results became visible, and makes no claim about it.** For SC-2, git chronology cannot settle the question: the commit introducing SC-2's definition is the same commit that introduces the first E3 result file. The project's account of SC-2's timing is contemporaneous and self-reported, and is not independent evidence.
- It does not establish whether the identification conditions I1–I4 were fixed before or after the three sources inspected on 2026-07-26. The repository's earliest artifact is dated 2026-07-27, and the conditions first appear that same day, so the only dated evidence runs the other way.
- It does not reclassify anything. Nothing was relabelled, removed or softened; the register enumerates, and the author decides what follows.

Signature

This statement is prepared by the agent and is **not** self-certified. Gate G7 carries the signature.

`

name:

role:

date:

statement:

`
