# Supplementary material for: When Can Orthodontic Over-Treatment Be Measured? A Pre-Registered Identification Assessment, a Falsified Claim, and a Cross-Sectional Analysis of Untreated Occlusal Need in NHANES III
Source: Healthcare (Basel). 2026 Oct 12;14(20):3395. doi: 10.3390/healthcare14203395 (PMC13704488; doi:10.3390/healthcare14203395)

Supplementary Figures

healthcare-4543715 · revision 1

Figure S1. Need and untreated occlusal need by age and sex. Post-hoc, descriptive.

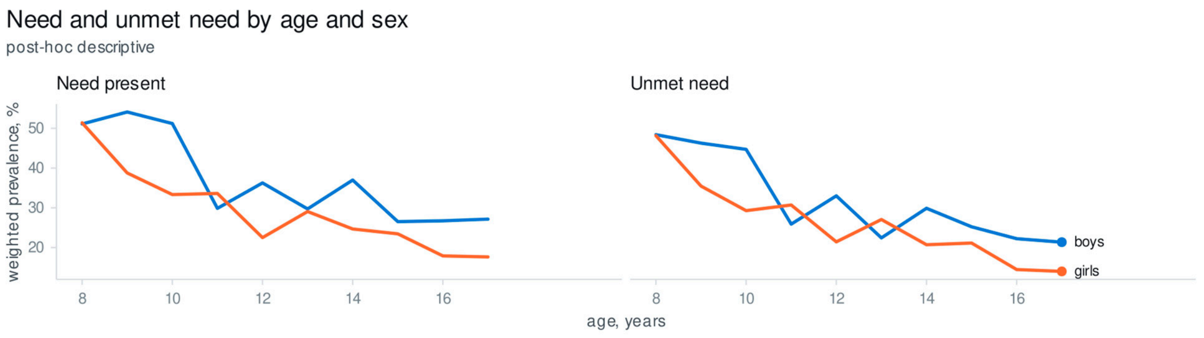

Figure S2. Completeness of the occlusal battery, showing how “could not be assessed” codes were handled.

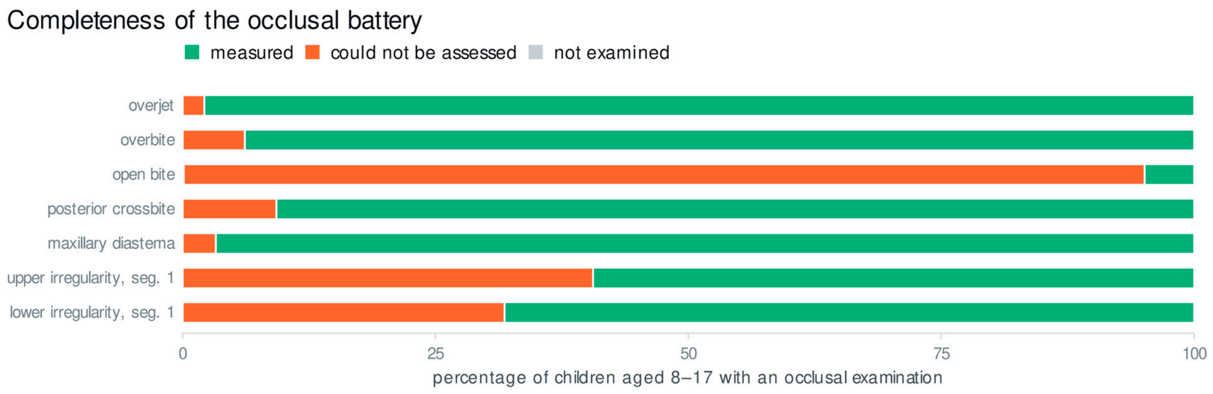

Open bite is recorded only when present: the survey manual establishes that positive overbite, negative overbite and open bite are mutually exclusive, so its high unassessable share reflects that structure rather than examination failure.
Sentinel codes never enter a numeric variable.

Figure S3. Prior against posterior for each regression parameter.

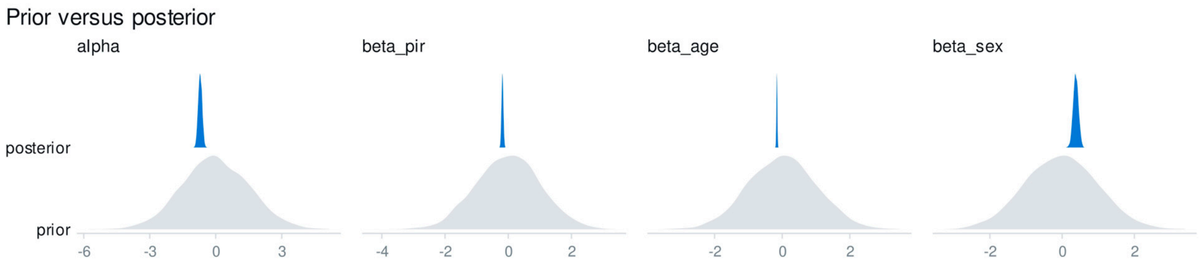

Priors were declared before fitting and are weakly informative on the logit scale. Each posterior is far narrower than its prior and none is concentrated at a prior boundary, so these estimates are determined by the data rather than by the prior.

Figure S4. The report-error bias analysis, post-hoc.

Differential report error does not explain the gradient away

95% credible interval for the unmet-need rank gradient under increasing differential under-report. Post-hoc, reviewer-requested.

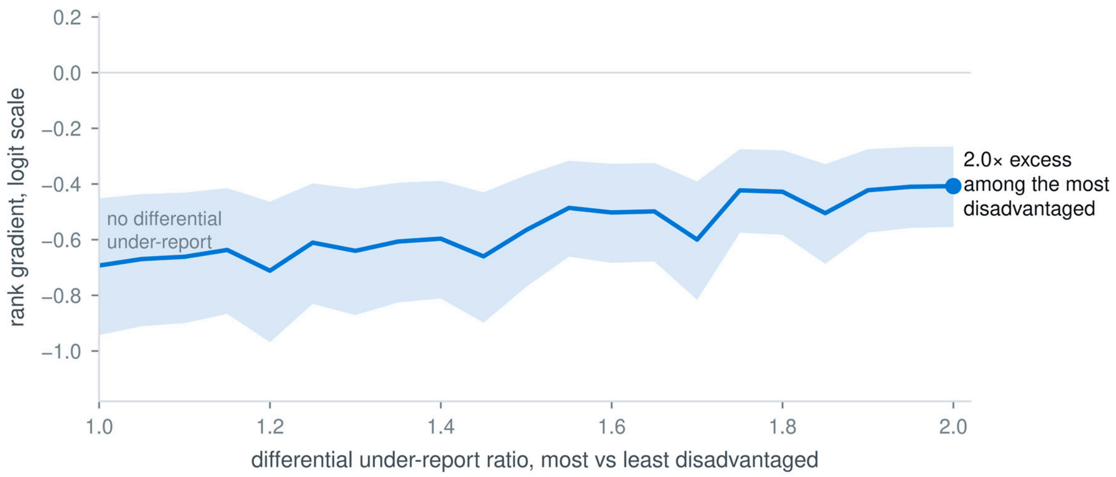

The interval excludes zero at every one of the 21 points examined (0 include zero), so there is no tipping point up to 2.0×. Non-differential under-report is not shown because it biases the association toward the null: correcting it makes the gradient stronger, not weaker.

Figure S5. The full recorded need composition of publicly funded starts in England and Wales.

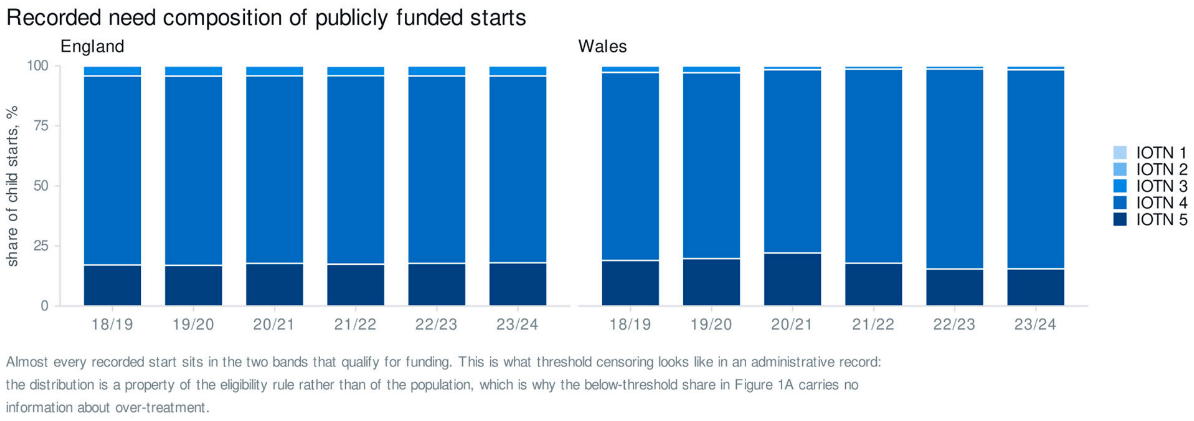

Figure S6. Posterior intervals and convergence diagnostics for every model parameter.

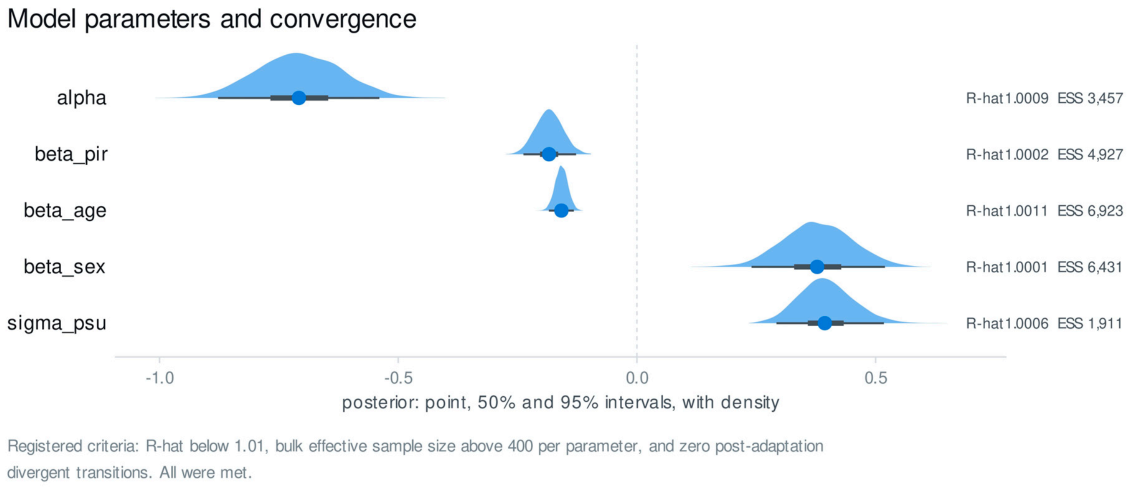

Figure S7. Posterior predictive check for the unmet-need model.

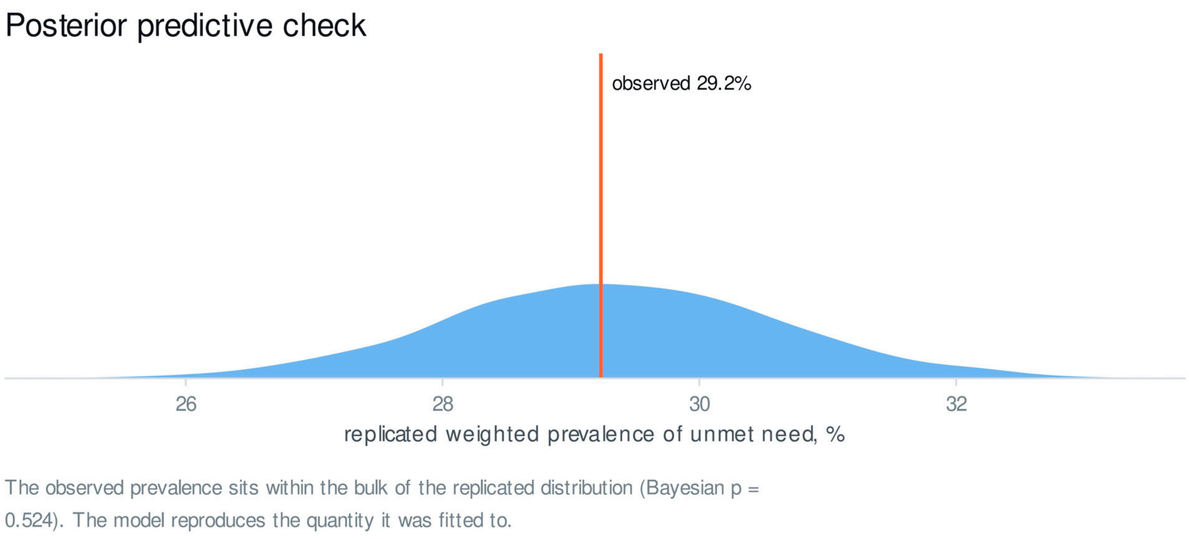

Figure S8. The registered contamination diagnostic.

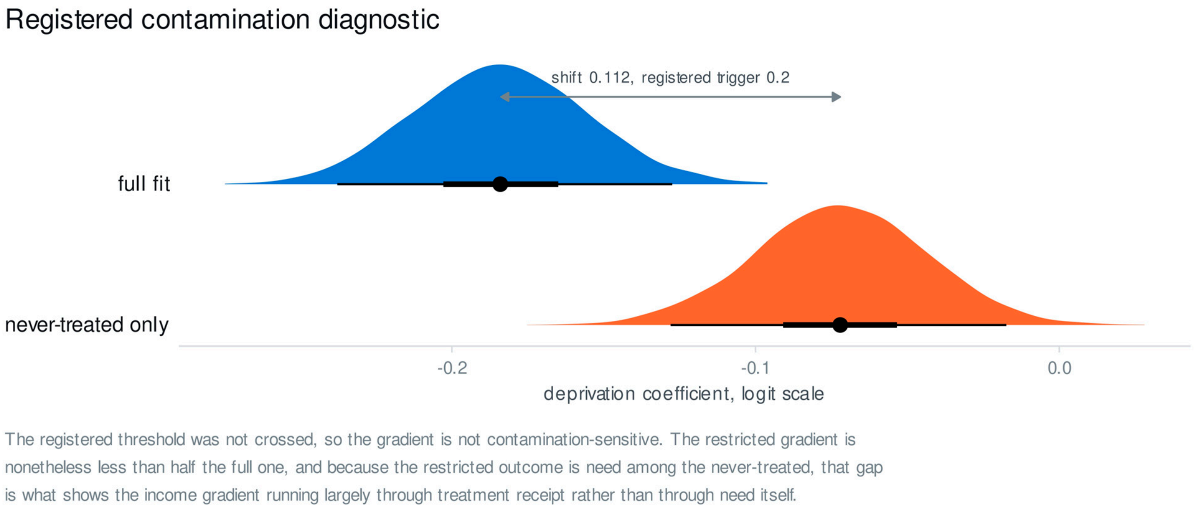

Figure S9. The rank gradient by age band, post-hoc.

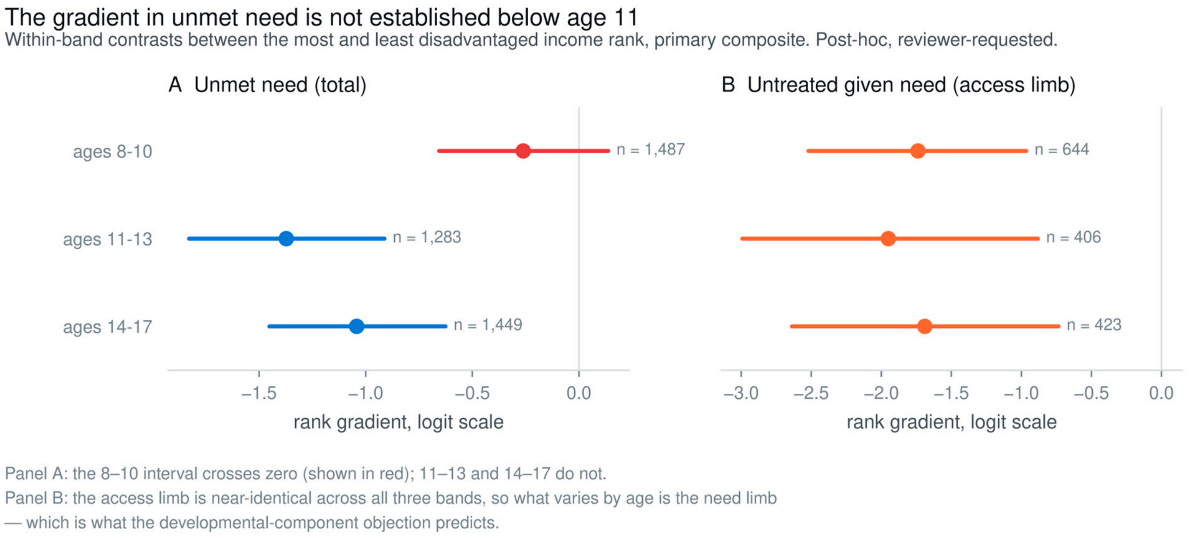

Supplement: Supplementary file 1 [file healthcare-14-03395-s001.zip › Supplementary Figures S1–S9.pdf]
