# Supplementary material for: When Can Orthodontic Over-Treatment Be Measured? A Pre-Registered Identification Assessment, a Falsified Claim, and a Cross-Sectional Analysis of Untreated Occlusal Need in NHANES III
Source: Healthcare (Basel). 2026 Oct 12;14(20):3395. doi: 10.3390/healthcare14203395 (PMC13704488; doi:10.3390/healthcare14203395)
Supplement: Supplementary file 1 [file healthcare-14-03395-s001.zip › Supplementary Tables S1–S5.pdf]

Supplementary Tables S1-S5

healthcare-4543715 · revision 2

Table S1. Every need component and all four composites by age band, post hoc.

row	age_band	n	n_positive	pct_unweighted	pct_weighted	ci_low	ci_high	registration_status
**N1_overjet_ge6mm**	all 8-17	4593	475	10.34	9.0	8.14	9.82	amendment_A01
**N1_overjet_ge6mm**	8-10	1605	188	11.71	11.04	9.63	12.36	amendment_A01
**N1_overjet_ge6mm**	11-13	1397	158	11.31	9.74	8.44	11.13	amendment_A01
**N1_overjet_ge6mm**	14-17	1591	129	8.11	6.88	5.73	8.11	amendment_A01
**N2_overbite_ge6mm**	all 8-17	4593	268	5.83	8.45	7.45	9.47	amendment_A01
**N2_overbite_ge6mm**	8-10	1605	89	5.55	8.13	6.29	9.99	amendment_A01
**N2_overbite_ge6mm**	11-13	1397	97	6.94	10.76	8.68	12.81	amendment_A01
**N2_overbite_ge6mm**	14-17	1591	82	5.15	6.95	5.54	8.34	amendment_A01
**N3_openbite_present**	all 8-17	4593	225	4.9	3.52	2.86	4.22	amendment_A01
**N3_openbite_present**	8-10	1605	86	5.36	4.24	3.02	5.47	amendment_A01
**N3_openbite_present**	11-13	1397	52	3.72	2.1	1.39	2.82	amendment_A01

**N3_openbite_present**	14-17	1591	87	5.47	4.05	2.83	5.26	amendment_A01
**N4_posterior_crossbite**	all 8-17	4593	334	7.27	7.35	6.4	8.27	amendment_A01
**N4_posterior_crossbite**	8-10	1605	93	5.79	7.09	5.1	9.05	amendment_A01
**N4_posterior_crossbite**	11-13	1397	95	6.8	6.28	4.81	7.76	amendment_A01
**N4_posterior_crossbite**	14-17	1591	146	9.18	8.34	7.01	9.64	amendment_A01
**N5_diastema_ge2mm**	all 8-17	4593	723	15.74	14.27	12.89	15.81	amendment_A01
**N5_diastema_ge2mm**	8-10	1605	423	26.36	30.28	27.12	33.5	amendment_A01
**N5_diastema_ge2mm**	11-13	1397	165	11.81	10.14	8.26	12.06	amendment_A01
**N5_diastema_ge2mm**	14-17	1591	135	8.49	5.13	4.28	6.01	amendment_A01
**anterior_irregularity_ge6mm**	all 8-17	4593	857	18.66	18.78	16.7	21.08	post_hoc_reviewer_requested
**anterior_irregularity_ge6mm**	8-10	1605	76	4.74	4.18	2.9	5.45	post_hoc_reviewer_requested
**anterior_irregularity_ge6mm**	11-13	1397	319	22.83	22.13	18.43	25.78	post_hoc_reviewer_requested
**anterior_irregularity_ge6mm**	14-17	1591	462	29.04	27.43	24.39	30.62	post_hoc_reviewer_requested
**composite_primary**	all 8-17	4593	1598	34.79	33.31	31.35	35.23	amendment_A01
**composite_primary**	8-10	1605	689	42.93	46.55	43.19	49.92	amendment_A01
**composite_primary**	11-13	1397	447	32.0	30.49	28.36	32.77	amendment_A01

**composite_primary**	14-17	1591	462	29.04	25.32	22.77	27.93	amendment_A01
**composite_SC1_with_irregularity**	all 8-17	4593	2107	45.87	44.45	42.26	46.75	amendment_A01
**composite_SC1_with_irregularity**	8-10	1605	723	45.05	48.15	44.85	51.35	amendment_A01
**composite_SC1_with_irregularity**	11-13	1397	637	45.6	43.3	39.9	46.92	amendment_A01
**composite_SC1_with_irregularity**	14-17	1591	747	46.95	42.47	39.47	45.7	amendment_A01
**composite_SC2_without_diastema**	all 8-17	4593	1099	23.93	23.43	21.98	24.91	post_hoc_other
**composite_SC2_without_diastema**	8-10	1605	394	24.55	25.79	22.8	28.57	post_hoc_other
**composite_SC2_without_diastema**	11-13	1397	341	24.41	23.45	21.15	25.78	post_hoc_other
**composite_SC2_without_diastema**	14-17	1591	364	22.88	21.61	19.35	23.86	post_hoc_other
**composite_SC3_no_diastema_with_irregularity**	all 8-17	4593	1665	36.25	35.63	33.58	37.71	post_hoc_reviewer_requested
**composite_SC3_no_diastema_with_irregularity**	8-10	1605	437	27.23	28.11	25.28	30.83	post_hoc_reviewer_requested
**composite_SC3_no_diastema_with_irregularity**	11-13	1397	553	39.58	38.08	34.9	41.45	post_hoc_reviewer_requested
**composite_SC3_no_diastema_with_irregularity**	14-17	1591	675	42.43	39.55	36.63	42.63	post_hoc_reviewer_requested

Table S2. **The need components and all four composites, with confidence intervals and a registration status on every row.**

row	n_positive	pct_unweighted	pct_weighted	ci_low	ci_high	n	registration_status
**N1_overjet_ge6mm**	475	10.34	9.0	8.14	9.82	4593	amendment_A01
**N2_overbite_ge6mm**	268	5.83	8.45	7.45	9.47	4593	amendment_A01
**N3_openbite_present**	225	4.9	3.52	2.86	4.22	4593	amendment_A01
**N4_posterior_crossbite**	334	7.27	7.35	6.4	8.27	4593	amendment_A01
**N5_diastema_ge2mm**	723	15.74	14.27	12.89	15.81	4593	amendment_A01
**composite_primary**	1598	34.79	33.31	31.35	35.23	4593	amendment_A01
**composite_SC1_with_irregularity**	2107	45.87	44.45	42.26	46.75	4593	amendment_A01
**composite_SC2_without_diastema**	1099	23.93	23.43	21.98	24.91	4593	post_hoc_other
**composite_SC3_no_diastema_with_irregularity**	1665	36.25	35.63	33.58	37.71	4593	post_hoc_reviewer_requested

Table S3. Included against excluded children, standardised mean differences, post hoc.

variable	type	included_pct	excluded_pct	included_mean	excluded_mean	smd
**age_years**	continuous			12.123	12.388	-0.0911
**sex = 1**	categorical	48.61	51.34			-0.0545
**sex = 2**	categorical	51.39	48.66			0.0545
**race_ethnicity (DMARETHN) = 1**	categorical	27.33	11.76			0.4003
**race_ethnicity (DMARETHN) = 2**	categorical	35.03	32.35			0.0567
**race_ethnicity (DMARETHN) = 3**	categorical	32.99	50.8			-0.367
**race_ethnicity (DMARETHN) = 4**	categorical	4.65	5.08			-0.0202
**race (DMARACER) = 1**	categorical	59.33	60.43			-0.0225
**race (DMARACER) = 2**	categorical	36.76	34.76			0.0418
**race (DMARACER) = 3**	categorical	3.91	4.81			-0.0442
**region (DMPCREGN) = 1**	categorical	11.35	10.43			0.0297
**region (DMPCREGN) = 2**	categorical	17.63	20.86			-0.0818
**region (DMPCREGN) = 3**	categorical	44.84	34.49			0.2128
**region (DMPCREGN) = 4**	categorical	26.17	34.22			-0.1762
**urbanicity (DMPMETRO) = 1**	categorical	47.62	54.55			-0.1389
**urbanicity (DMPMETRO) = 2**	categorical	52.38	45.45			0.1389
**design stratum (SDPSTRA6, proxy) [worst level 48]**	categorical	None	None			-0.2901
**component N1_overjet_ge6mm**	binary	10.48	8.82			0.056
**component N2_overbite_ge6mm**	binary	5.9	5.08			0.0361

**component N3_openbite_present**	binary	4.84	5.61		-0.035
**component N4_posterior_crossbite**	binary	7.21	8.02		-0.0308
**component N5_diastema_ge2mm**	binary	15.98	13.1		0.0816
**anterior irregularity >= 6mm**	binary	18.58	19.52		-0.0238
**composite need present**	binary	34.91	33.42		0.0314
**never treated (prior_treatment=excluded)**	binary	89.17	91.98		-0.0963
**treatment receipt stated**	binary	99.57	99.73		-0.0271

Table S4. The full gradient analysis under SC-2 and SC-3, post hoc.

composite	outcome	n	risk_most_disadvantaged_pct	risk_p0_ci_low	risk_p0_ci_high	risk_most_advantaged_pct	risk_p1_ci_low	risk_p1_ci_high	risk_difference_pp	rd_ci_low	rd_ci_high	rii	rii_ci_low	rii_ci_high	beta_rank_logit	beta_ci_low	beta_ci_high	max_rhat	min_ess_bulk	divergences	registration_status
**Registered primary**	total unmet need	4219	37.62	34.73	40.7	21.71	19.39	24.25	15.91	11.15	20.65	1.741	1.464	2.053	-0.838	-1.093	-0.582	1.0363	1421.55	0	registered
**Registered primary**	need limb	4219	39.21	36.29	42.17	27.73	25.08	30.44	11.48	6.56	16.4	1.419	1.218	1.647	-0.563	-0.808	-0.32	1.0239	1811.16	0	post_hoc_other
**Registered primary**	access limb	1473	94.66	92.59	96.31	78.03	73.28	82.48	16.63	10.91	22.47	1.214	1.133	1.307	-1.793	-2.4	-1.177	1.0214	1432.42	0	post_hoc_other
**SC-2 (no diastema)**	total unmet need	4219	26.53	23.74	29.54	13.98	12.06	16.11	12.56	8.19	17.03	1.914	1.519	2.39	-0.825	-1.123	-0.538	1.0353	1820.2	0	post_hoc_reviewer_requested

**SC-2 (no diastema)**	need limb	4219	28.17	25.38	31.07	18.7	16.47	21.13	9.48	4.9	13.92	1.515	1.235	1.834	-0.554	-0.818	-0.285	1.0253	1919.7	0	post_hoc_reviewer_requested
**SC-2 (no diastema)**	access limb	1006	92.49	89.47	94.9	74.9	69.3	80.07	17.6	10.31	24.66	1.237	1.13	1.355	-1.672	-2.353	-0.978	1.0115	1358.2	0	post_hoc_reviewer_requested
**SC-3 (no diastema, + irregularity)**	total unmet need	4219	42.16	39.02	45.21	19.7	17.48	22.03	22.46	17.63	27.07	2.15	1.808	2.527	-1.144	-1.39	-0.894	1.0563	1860.4	0	post_hoc_reviewer_requested
**SC-3 (no diastema, + irregularity)**	need limb	4219	44.1	41.03	47.16	27.48	24.88	30.09	16.62	11.59	21.56	1.61	1.386	1.862	-0.785	-1.031	-0.545	1.0229	1642.4	0	post_hoc_reviewer_requested
**SC-3 (no diastema, + irregularity)**	access limb	1526	93.59	91.4	95.39	72.69	67.93	77.35	20.9	14.71	26.84	1.289	1.19	1.395	-1.935	-2.496	-1.368	1.0171	1309.6	0	post_hoc_reviewer_requested

Table S5. Design-based against Bayesian estimation, NHANES III children aged 8–17 examined in 1988–1994.

Quantity	Bayesian estimate	Design-based estimate	Taylor SE	Taylor 95% CI	Replicate SE	Replicate 95% CI	SE ratio	Superseded SE
Need prevalence, weighted %	33.31	33.31	1.380	30.61 to 36.02	1.137	31.08 to 35.54	0.82	5.671
Untreated occlusal need, weighted %	29.21	29.23	1.186	26.91 to 31.56	0.938	27.40 to 31.07	0.79	4.662
Never treated among those with need, weighted %	87.80	87.75	1.354	85.10 to 90.41	1.214	85.38 to 90.13	0.90	—
Untreated occlusal need, PIR quintile 1 (most disadvantaged), %	—	33.27	1.708	29.93 to 36.62	1.789	29.77 to 36.78	1.05	—
Untreated occlusal need, PIR quintile 2, %	—	34.01	2.208	29.68 to 38.34	1.829	30.43 to 37.60	0.83	—
Untreated occlusal need, PIR quintile 3, %	—	35.52	2.343	30.93 to 40.11	2.328	30.96 to 40.08	0.99	—
Untreated occlusal need, PIR quintile 4, %	—	24.77	2.239	20.38 to 29.16	1.765	21.31 to 28.23	0.79	—
Untreated occlusal need, PIR quintile 5 (most advantaged), %	—	18.97	2.540	13.99 to 23.94	2.165	14.72 to 23.21	0.85	—
Rank gradient, logit scale	−0.838	−0.861	0.154	−1.171 to −0.551	0.142	−1.147 to −0.576	0.92	—

CI, confidence interval; PIR, poverty income ratio; SE, standard error. Percentages are weighted to the US non-institutionalised population using the survey examination weight WTPFEX6; the rank gradient is on the logit scale and is not a percentage. Every quantity in this table is reviewer-requested and post-hoc, declared in advance in registered amendment A-02.

Estimation. Taylor estimates use Taylor-series linearisation on the survey's own stratum, primary sampling unit and examination weight variables. Replicate estimates use the 52 replicate weights the survey ships, under the specification NCHS documents for them: Fay's method with K = 0.3 and the variance estimator $v(\theta) = 1/[G(1-K)^2] \times \Sigma(\theta_j - \theta)^2$, summing squared deviations about the full-sample estimate. The corrected variance was verified to six decimal places against a hand computation before use. Point estimates are identical between the two design-based routes by construction, to within 5×10^{-4} on every quantity; the Taylor column is reported because it involves no replication-scheme parameter, and the replicate column is the independent crosscheck. Bayesian estimates are posterior means from the design-informed hierarchical logistic model of Section 2.8 and are reported where the model produces the corresponding quantity.

The superseded column. The final column reports the replicate standard error as it stood before correction, and is retained so that the correction is visible rather than taken on trust. Two errors were present in the original crosscheck. The replicate design had been declared with a jackknife multiplier of $(G-1)/G = 0.980769$ rather than the documented Fay multiplier of 0.0392465, a ratio of 5.00 on the standard-error scale; and squared deviations were being summed about the mean of the replicate estimates rather than about the full-sample estimate.

Because a variance multiplier cannot move a point estimate, the fault was visible as an inflation of the standard errors alone while the point estimates reproduced exactly. The original run covered two quantities with recorded standard errors, which is why the column is otherwise empty. Superseded values remain in the archived record and are not overwritten.

Agreement. Under the documented specification, replicate standard errors run between 0.79 and 1.05 times the Taylor ones across all nine quantities, against 4.11 on need prevalence before the correction. The two routes are not expected to agree exactly: the replicate weights carry the survey's nonresponse and poststratification adjustments, which Taylor linearisation treats as fixed, and that generally lowers the replicate variance. The residual difference is of that kind.
